# Hindlimb biomechanics of *Lagosuchus talampayensis* (Archosauria, Dinosauriformes), with comments on skeletal morphology

**DOI:** 10.1111/joa.14183

**Published:** 2024-12-04

**Authors:** Alejandro Otero, Peter J. Bishop, John R. Hutchinson

**Affiliations:** ^1^ CONICET División Paleontología de Vertebrados (Anexo Laboratorios) La Plata Argentina; ^2^ Structure and Motion Laboratory, Department of Comparative Biomedical Sciences Royal Veterinary College North Mymms Hatfield UK; ^3^ Museum of Comparative Zoology and Department of Organismic and Evolutionary Biology Harvard University Cambridge Massachusetts USA; ^4^ Geosciences Program Queensland Museum Brisbane Queensland Australia

**Keywords:** archosaur, dinosaur, locomotion, muscle, osteology, palaeontology

## Abstract

*Lagosuchus talampayensis* is a small‐bodied (~0.5 m long) Late Triassic dinosauriform archosaur from Argentina. *Lagosuchus* long has been a pivotal taxon for reconstructing the evolution of form and function on the dinosaur lineage. This importance is because it has a mix of ancestral archosaurian traits, such as a small pelvis with a mostly closed acetabulum lacking prominences that would restrict hip mobility much, with derived “dinosaurian” traits such as bipedalism, proximally shifted thigh muscle insertions, elongate hindlimbs, “advanced mesotarsal” ankle joints and digitigrade feet. Here, to quantify key functional traits related to the locomotor biomechanics of *Lagosuchus*, we build a three‐dimensional musculoskeletal model, focussing on morphofunctional analysis of the pelvic limb. We survey skeletal material that we have digitised, pointing out hitherto undescribed features and elements, many of which are from taxa other than *Lagosuchus*. Next, we select ideal elements amongst these to construct a composite model, and articulate adjacent body segments into joints, then estimate body shape including centre of mass, and add muscle paths to create a musculoskeletal model. Finally, we use two methods to quantify the hindlimb muscle parameters (“architecture”) in the model. We find that they produce similar estimates of force‐generating capacities, and compare these data to the few available data from other archosaurs in an evolutionary context, to reconstruct fundamental patterns of changes in muscle architecture and pelvic limb morphology. Our model forms a valuable basis for future quantitative analyses of locomotor function and its evolution in early archosaurs, and an example of how to navigate decision‐making for modelling problematic specimens.

## Graphical abstract

The study constructs a 3D whole‐body biomechanical model of the Late Triassic archosaurian reptile Lagosuchus talampayensis, which includes reconstructions of the hindlimb musculature. The process of constructing the model revealed that numerous limb bones curated with the main specimens are from other, as‐yet‐undetermined archosaurs. The model's muscle sizes and strengths are estimated via two techniques and compared, showing broadly similar results. The data obtained are placed into a phylogenetic context, revealing preliminary estimates of how skeletal changes relatec to changes of muscle sizes across Archosauria.

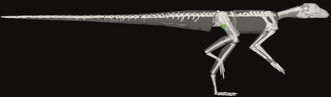

## 1 INTRODUCTION

**FIGURE 1:**
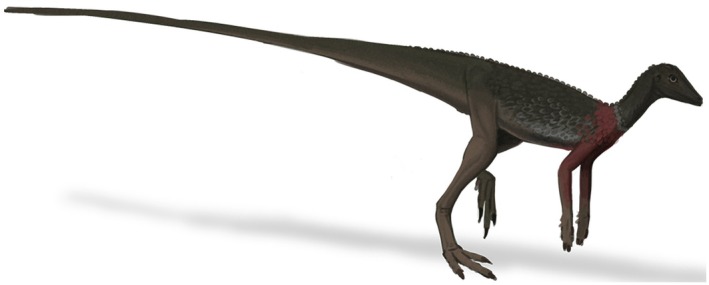
Artistic reconstruction of *Lagosuchus talampayensis*, based on published dimensions; here purely used for visualisation purposes. By John Conway. Total body length approximately 50 cm.

A taxon originally called *Lagosuchus talampayensis* by Romer (1971; also see Romer, 1972; Bonaparte, 1975; including the second species *L. lilloensis*) was a small dinosauriform archosaur from the early Carnian (early Late Triassic) of Argentina (Marsicano et al., 2016). *Lagosuchus* is extremely important for understanding the early evolution of dinosauriforms and other avemetatarsalian archosaurs, as well as the origin of Dinosauria (e.g., Novas, 1996), because its general body plan (Figure 1), erect hindlimb posture and habitual bipedalism are traits expected for an ancestral dinosauriform (e.g., Allen et al., 2013, 2021; Carrano, 2000; Charig, 1972; Fechner, 2009; Grinham et al., 2019; Hutchinson & Gatesy, 2000; Kubo & Kubo, 2012; Langer et al., 2013; Novas, 1992; Padian, 2012; Sereno & Arcucci, 1994). Furthermore, ornithodirans (specifically, pterosaurs and dinosaurs) survived the Triassic‐Jurassic transition, and locomotor abilities of these taxa have sometimes been advocated to explain this survival (vs. extinction of most other archosaur clades; reviewed by Cuff et al. 2022, and see also Shipley et al, 2024). Thus understanding locomotor function in early ornithodirans such as *Lagosuchus* has the potential to provide important insight in understanding that survival.

Sereno and Arcucci (1994) deemed the somewhat fragmentary holotype material of *Lagosuchus talampayensis* to be non‐diagnostic and thus renamed it *Marasuchus lilloensis*. More recently, Agnolín and Ezcurra (2019) re‐examined the relevant specimens and argued that there were diagnostic features overlapping with some of those in other specimens such as the holotype of “*M. lilloensis”*, thus resurrecting *Lagosuchus talampayensis*. Nonetheless, these features are few, and many key traits of the genus and its two species are not preserved in the holotype, so the issue remains complex. The broader clade Lagosuchidae includes the two known species, but other fragmentary specimens such as *Saltopus elginensis*, from the Late Triassic of Elgin, Scotland, might also be included, or other dinosauromorphs (e.g., Benton & Walker, 2011).

Here we primarily are interested in reconstructing the musculoskeletal function of the pelvic limb apparatus in *Lagosuchus*. Despite its importance, there are few prior functional studies of this taxon; most studies (cited above) have focused on osteological description and phylogenetic analyses. Remes (2008) and Fechner (2009) studied *Lagosuchus* in the context of archosaurian forelimb and hindlimb muscular evolution, respectively. Allen et al. (2013) constructed a spline‐based three‐dimensional (3D) volumetric model of “*Marasuchus lilloensis”* using a cast seemingly based mainly on that taxon's holotype as well as a reconstructed sculpture of unpreserved regions in the tail, distal forelimbs and skull. They used this model to reconstruct the centre of mass and other body segment parameters, and estimate its caudofemoralis muscle mass, and, along with similar models of other taxa, to reconstruct the evolution of those traits across Archosauria, especially within theropod dinosaurs. Bishop et al. (2020) used this model in a morphometric (statistical) analysis of body shape to estimate which archosaurs might have been quadrupedal or bipedal, supporting the inference of its bipedal habits. Allen et al. (2021) and Cuff et al. (2022) used the underlying skeletal model to build a 3D musculoskeletal model in order to quantify muscle moment arms (actions, or leverages), again across archosaurian phylogeny, with a focus on Theropoda. Pintore et al. (2021) included 3D shape data of the femur of “*L. lilloensis”* (specimen PVL 3871; reported as PVL 4670; see below) in a morphometric analysis across Archosauriformes, recurrently finding that it was most similar to other bipedal taxa. In contrast to this apparent consensus, Gonet et al. (2023) used micro‐computed tomography (micro‐CT) scans of the femur of “*M. lilloensis”* in another morphometric study, here focussing on geometric and microanatomical parameters of cross‐sections in Reptilia, and surprisingly found that its morphology classified as a facultative biped with a sprawling hindlimb posture, although they considered this result dubious in light of the studies described above. Furthermore, some studies have reassessed the functional morphology of ornithodirans such as *Lagosuchus* and considered the possibility that limb postures in some taxa were more sprawling than erect and (from concluding that no diagnostic forelimb material may exist; and outgroup comparisons) perhaps quadrupedal (e.g., Agnolín et al., 2024; Fechner, 2009; Gônet et al., 2023; Piechowski & Tałanda, 2020; Remes, 2008). A revisited appraisal of *Lagosuchus* specimens and their functional significance is therefore quite timely.

Our reassessment of musculoskeletal anatomy and locomotor function follows methods for 3D modelling similar to those applied to the theropod *Coelophysis bauri* by Bishop, Cuff, and Hutchinson (2021). Our five aims are to: (1) Describe the appendicular (and, more briefly, caudal vertebral and cranial) osteology of *Lagosuchus talampayensis* from original specimens and micro‐CT scans, attempting to clarify which specimens currently ascribed to or curated with that taxon definitely pertain to it; (2) With some of those specimens, construct a composite 3D skeletal model with joints connecting skeletal elements; (3) Reconstruct the dimensions of all major body segments (mass, centre of mass) and compare the results with those of prior studies; (4) Attempt a new reconstruction of the hindlimb musculature and architecture and integrate it into a 3D musculoskeletal model; and (5) Use these new data to estimate the maximal muscle forces and “antigravity” joint moments the animal might have been able to generate, how these estimates depend on which methods are used to reconstruct detailed muscle architecture, and how they compare with those for other early (and extant) archosaurian taxa reconstructed to date. Wherever pertinent, we present and discuss our results in a phylogenetic context (e.g., see Ezcurra, Nesbitt, Bronzati, et al., 2020; Gauthier, 1986; Langer et al., 2013; Novas, 1992,2010, 2011; Sereno, 1991).

## 2 MATERIALS AND METHODS

### 2.1 Specimens, scanning and segmentation

We studied specimens as listed in Table 1, using the original specimens and micro‐CT scans with parameters listed in that table. Museum collection abbreviations are: MCZ (Museum of Comparative Zoology, Harvard University, Cambridge, MA, USA), NHMUK PV (Natural History Museum, London, United Kingdom), PULR (Paleontología, Museo de Ciencias Naturales, Universidad Nacional de La Rioja, La Rioja, Argentina) and PVL (Colección Paleontología de Vertebrados, Facultad de Ciencias Naturales e Instituto Miguel Lillo, Universidad Nacional de Tucumán, Tucumán, Argentina). We inspected micro‐CT data for the left hindlimb of the *Lagosuchus talampayensis* holotype PULR 09, but do not describe that here. Likewise, we did not study MCZ 4137 (ex 4116) but note that this specimen badly needs proper description in light of confusion about taxonomic assignments of specimens (see Discussion). We semi‐manually segmented the scan data in Mimics (version 21; Materialise, Inc.; Leuven, Belgium) software, exporting polygonal meshes of bones as STL files.

**TABLE 1:** Specimens that were focused on by key studies of *Lagosuchus* and “*Marasuchus*”; and in this study (or mentioned in the Introduction).

Material described	Romer, 1971, 1972	Bonaparte, 1975	Sereno & Arcucci, 1994	Agnolín & Ezcurra, 2019
PVL 3870		x	x	x
PVL 3871 (holotype of “*Marasuchus lilloensis*”)	x	x	x	x
PVL 3872		x	x	x
PVL 4670			x	“absence of autapomorphies or a unique combination of character states that may support their species level assignment” (p. 6)
PVL 4671			x	x
PVL 4672			x (but no mention of forelimbs)	x
PULR 09 (holotype of *Lagosuchus talampayensis*, ex MLP 64‐XI‐14‐11)	x	x	x	x
NHMUK PV R14101 (ex BMNH R14101)				
MCZ 4137 (ex 4116)	x	x		x

### 2.2 Composite model

**FIGURE 2:**
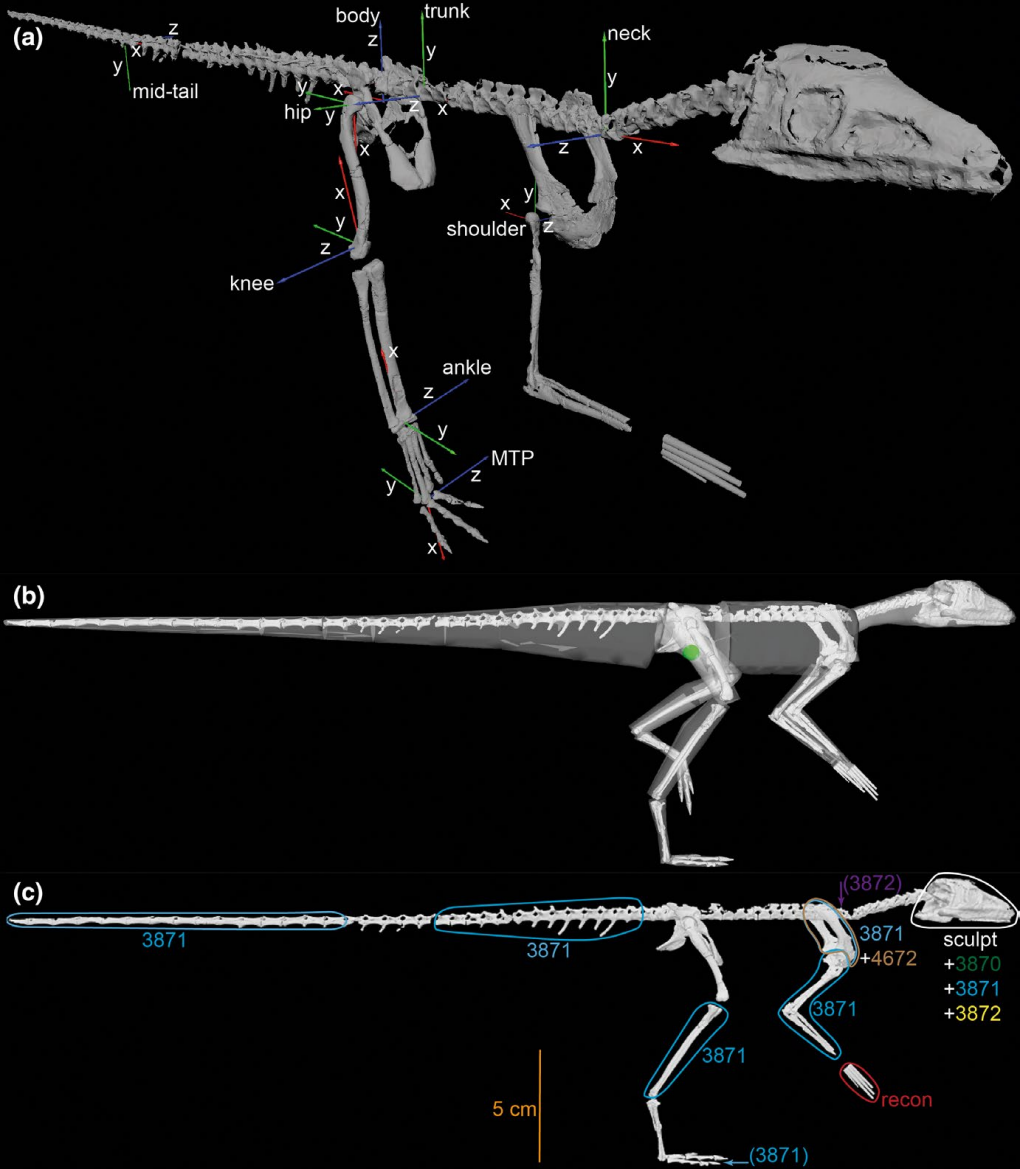
Whole‐body model of *Lagosuchus*. (a), Major joint coordinate systems (JCSs), focussed on the right side of the body; in oblique craniolateral view; in the reference pose (all joint angles = 0°). (b), “fleshed‐out” body used to quantify body segment parameters (BSPs), with the body's centre of mass (COM; when in reference pose with hips abducted 15°) shown as a green sphere below the hips; in right lateral view; posed in the limb orientation used for estimating maximal muscle moments (see below). (c) skeleton‐only image with scale bar and specimen sources (also see Table 2) labelled alongside circled elements. Joints in (a) are labelled (proximal tail is not shown); axes follow (red, green, blue) = (x, y, z), employing the same conventions used to describe joint disposition as outlined by Gatesy et al. (2022). Elements not circled and labelled in (c) are from PVL 3870. “MTP” in (a) = (third) metatarsophalangeal joint. “recon” in (c) = our reconstruction of the manus (see Methods text); “sculpt” refers to the NHMUK PV R14101 cast/sculpture. (a) and (b) are not to scale.

For the descriptive and comparative first aim of the study, we inspected the original scan and segmentation data as well as the bone meshes (in Meshlab v2022 software; Cignoni et al., 2008; https://www.meshlab.net/). We then selected the ideal bones from those specimens to construct our 3D composite skeletal model for our second aim, using PVL 3870 as the focal specimen, as its pelvic appendicular skeletal elements are best‐preserved. Table 2 lists which different elements from which specimens were used for bones in the model, achieving our Aim 2. Our protocol from here onwards followed that of Bishop, Cuff, and Hutchinson (2021), so we describe our methods more briefly. To connect these bones via joints in 3D, we manually articulated them in Meshlab and then saved the bones as OBJ files. As there is no manus (or carpus) known for *Lagosuchus*, we estimated the general morphology from the reconstruction in Sereno and Arcucci (1994) and the dimensions of NHMUK PV R14101 (Figure S1), using simple cylinders for digits (Figure 2a); deviations from this would have negligible impact on the results we discuss here.

**TABLE 2:** Specimen numbers of skeletal elements used to build the composite 3D model. Elements were scaled linearly to the proportions of PVL 3870, with scaling factors shown in brackets atop each column.

Specimen	PVL 3870	PV 3871 [0.72]	PVL 3872 [1.3]	PVL 4670	PVL 4671	PVL 4672 [1.0]	NHMUK PV R14101 [0.73]
Element(s)							
Maxilla	R						
Jaw joint		R					
Basicranium	X		X				
Skull							X
Cervicals	1–9						
Dorsals	6–15		1–5				
Proximal caudals	1–2	3–10					
Middle caudals	11–15	16–20					
Distal caudals		21–35					
Scapulocoracoid		L				R	
Humerus		L					
Radius		L					
Ulna		L					
Manus							R
Pelvis‐sacrum	L						
Femur	L, R						
Tibiotarsus		L					
Fibula		L					
Tarsometatarsus	L, R						
Pedal phalanges	D1‐3 L, D4 R	D4 p4 L					
Unguals	p1‐3 R	3? L					

Abbreviations: D, digit(s); L, left; p, phalanx/phalanges; R, right; X, skull parts used in model; ? = uncertain identification of side or numbering.

Those OBJ mesh files were duplicated and further edited in Meshlab to isolate the articular surfaces of major hindlimb joints (acetabulum and proximal and distal femur, tibiotarsus, and ‘tarsometatarsus’) as well as the glenoid and proximal humerus, then the centra of two sacral vertebrae and appropriate centra to form four intervertebral joints (proximal tail, midtail, trunk above the pubis, and base of the neck just cranial to the pectoral girdle). We imported these OBJ files into the custom MATLAB (v9.5; The Mathworks, Inc., Natick, MA, USA) script from Bishop, Cuff, and Hutchinson (2021) to fit geometric primitives to these meshes, using planes for proximal ends of limb bones, spheres for the acetabulum and glenoid, a sphere for the proximal femur, and cylinders for the distal ‘tibiotarsus’ and ‘tarsometatarsus’ as well as the sacrum and intervertebral joints (see also Gatesy et al., 2022). The metatarsophalangeal (MTP) joint was simplified to focus only on the third joint of the third digit, and we did not include interphalangeal motions. The coordinates of all joints were then used, along with the imported bone files, in Rhinoceros software (v5; Robert McNeel & Associates, Seattle, WA, USA) to articulate the skeleton with anatomical coordinate systems (ACSs) that would ultimately determine joint coordinate systems (JCSs) and joint centres of rotation; following Gatesy et al. (2022) and Bishop, Cuff, and Hutchinson (2021); with space of 5% femur length added between the femur and tibiotarsus following Hutchinson et al. (2005), Holliday et al. (2010) and Bishop, Cuff, and Hutchinson (2021). Those ACS 3D coordinates were reconstructed in Maya software (v2022; Autodesk, Inc.; San Francisco, CA, USA) to export OBJ meshes representing the ACSs (used later; below). As per Bishop, Falisse, et al. (2021), we simplified the modelling of the forelimb as only having a shoulder (glenohumeral) joint, with the elbow posed at 90° (forearm vs. upper arm angle; Figure 2a). Again, due to the small mass of the forelimb, the exact pose used would have minimal effect on our hindlimb‐focussed results.

For achieving our Aim 3, a series of octagonal ‘hoops’ (4 radial, 4 diagonal points) were fit around sections of the skeleton in frontal view (axial skeleton) and proximodistal cross‐sections (limbs) to define outlines of the soft tissues; following Bishop, Cuff, and Hutchinson's (2021) modified version of Allen et al.'s (2009, 2013) and Hutchinson et al.'s (2011) methodology. An abbreviated description follows. An initial model with hoop points positioned by skeletal landmarks (where present; or along lines connecting landmarks, including lines of action of key muscles used in the musculoskeletal model; see below) was created for further analysis. An ellipsoid was fit between the inner surfaces of the dorsal vertebrae, scapulocoracoid and pelvis to represent the (missing) ribcage, with its width approximating the lateral margins of the girdle elements, and hoop points matched to the perimeter of these skeletal and ellipse points. Because this ellipsoid was speculative, we added a sensitivity analysis of the final trunk segment in which mass was increased by 20% and the resulting whole‐body COM was calculated in OpenSim's Analysis: BodyKinematics tool. Air spaces inside the skull approximated sinus and pharynx geometry, and lungs used a “crocodile” shape (Allen et al., 2009) confined to the cranial half of the trunk segment (not a large ‘bird‐like’ lung system; following Schachner et al., 2011, 2013). Tail hoops followed the method of Allen et al. (2009) based on extant saurian skeleton‐to‐flesh geometry. We then used a mean of the maximal (all 8 points inflated 20% then diagonal points inflated 20.7% more; ‘tracheal’ air space deflated 20%) and minimal (neck, limbs and tail hoops deflated 20% then diagonal points deflated 14.3% more; ‘tracheal’ air space inflated 20%) hoops as the baseline model; but we did not deflate/inflate head, manus, pes or digits segments. Mean hoops then were lofted together to produce ‘watertight’ meshes. The resulting OBJ mesh files of bones and soft tissue geometry (for body segment parameters; BSPs) then were processed via a custom MATLAB script; again following Bishop, Cuff, and Hutchinson's (2021) modified version of Allen et al.'s (2013) methodology after converting them to triangular meshes. Final meshes are in the associated data. Body segment densities were assumed to have a density of 1000 kg m^−^^3^, but we included zero‐density cavities that we reconstructed in Rhinoceros for air spaces as above. Prior studies have shown the assumptions about air space dimensions to have small effects (e.g., Allen et al., 2009; Durston et al., 2022; Macaulay et al., 2017) so we did not investigate the issue further. The MATLAB script processed the ACSs, bone and soft tissue OBJs to produce a whole‐body model with JCSs and BSPs as per Allen et al. (2013), Bishop, Cuff, and Hutchinson (2021), and Gatesy et al. (2022), in OpenSim v4.5's. osim format (Figure 2) (https://simtk.org/projects/opensim/; Delp et al., 2007; Seth et al., 2018). We do not report inertial tensor values (other BSPs) here, but discuss masses, lengths and COMs later as part of Aim 3.

**FIGURE 3:**
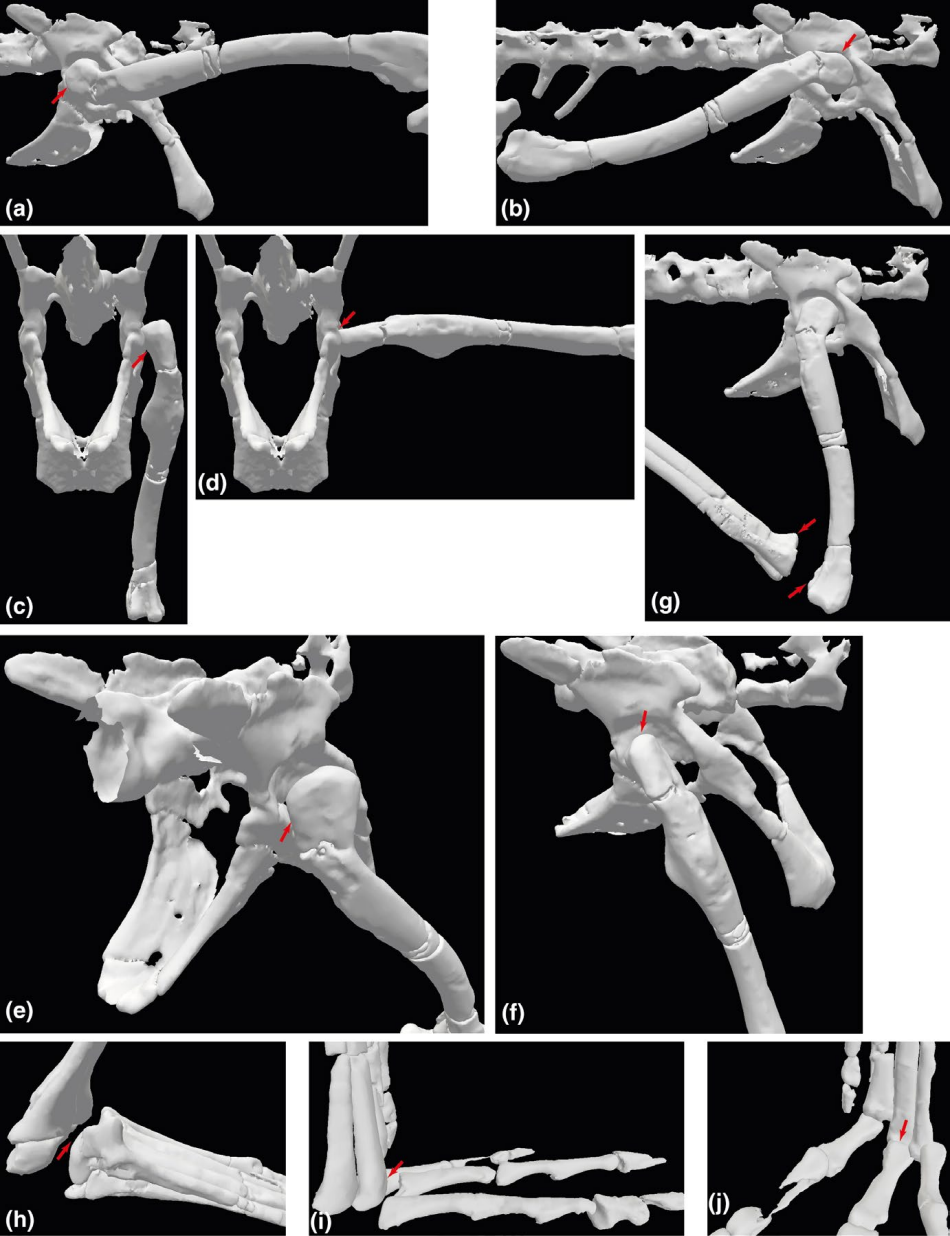
Maximal and minimal joint poses interpreted for the model of *Lagosuchus*; used to calculate ROMs (in Table 3). Hip flexion (a) and extension (b) in lateral view; adduction (c) and abduction (d) in caudal view, external long‐axis rotation in caudolateral view (e), internal long‐axis rotation in craniolateral view (f), knee flexion in lateral view (g), ankle (dorsi)flexion in lateral view (h), MTP joint dorsiflexion in lateral view (i) and MTP joint plantarflexion in caudolateral view (j). Red arrows indicate articular interactions used to infer ROM limits.

We used the musculoskeletal model to conduct three main objectives. For Objective 1 (related to Aim 2; adding joints connecting skeletal elements), we estimated joint minimal/maximal angles and thereby ranges of motion (ROMs) around each degree of freedom (DOF) by inspecting bone‐on‐bone collision or disarticulation in OpenSim (Figure 3). We did not quantify ROMs more precisely than 5° intervals. Table 3 shows the ROM endpoints for each joint (except 0° endpoints such as for the knee and ankle; see Figure 2a); three DOFs were allowed for the hip and shoulder, two for intervertebral joints (dorsal/ventral flexion and lateral flexion), and one for all other limb joints. These relatively basic DOFs (e.g., no translations) certainly were a simplification vs. actual joint function (e.g., Bishop et al., 2023; Demuth et al., 2020; Kambic et al., 2014; Manafzadeh et al., 2021, 2024; Manafzadeh & Gatesy, 2021). This assumption was deemed necessary for the modelling work we conducted here because basic ROMs are needed in the model but our study does not investigate maximal joint mobility volumes as per the latter studies. Intervertebral and shoulder joint ROMs were input into the model as arbitrary values (e.g., −30° to 30°) because they are not a focus of this study (see also Bishop, Falisse, et al., 2021). We used a custom script (via Lars d'Hondt, KU‐Leuven, Belgium; yet to be published) to mirror the right side to the left side for the final model, ensuring precise mediolateral symmetry of all BSPs, JCSs, muscles, wrapping objects and other key inputs.

**TABLE 3:** Degrees of freedom (DOF) and ranges of motion (ROM) allowed for joints; in degrees; in the model of *Lagosuchus*. “Min” and “Max” are minimum and maximum angles; and like ROM are in degrees.

Joint	DOF	Min	Max	ROM
Hip	flex/ext	−90	75	165
	add/abd	0	90	90
	int/ext. rot	−90	50	140
Knee	ext/flex	0	130	130
Ankle	flex/ext	−100	0	100
MTP3	flex/ext	−180	0	180
Shoulder	flex/ext	−90	90	180
Proximal tail	DV flex/ext	−30	30	60
	flex (lateral)	−30	30	60
Mid‐tail	DV flex/ext	−30	30	60
	flex (lateral)	−30	30	60
Trunk	DV flex/ext	0	30	60
	flex (lateral)	−30	30	60
Neck base	DV flex/ext	−30	30	60
	flex (lateral)	−30	30	60

Abbreviations: add/abd, adduction/abduction; DV, dorsoventral; MTP3, third metatarsophalangeal joint; flex/ext, flexion/extension; int/ext, internal (medial)/external (lateral) long‐axis rotation.

**FIGURE 4:**
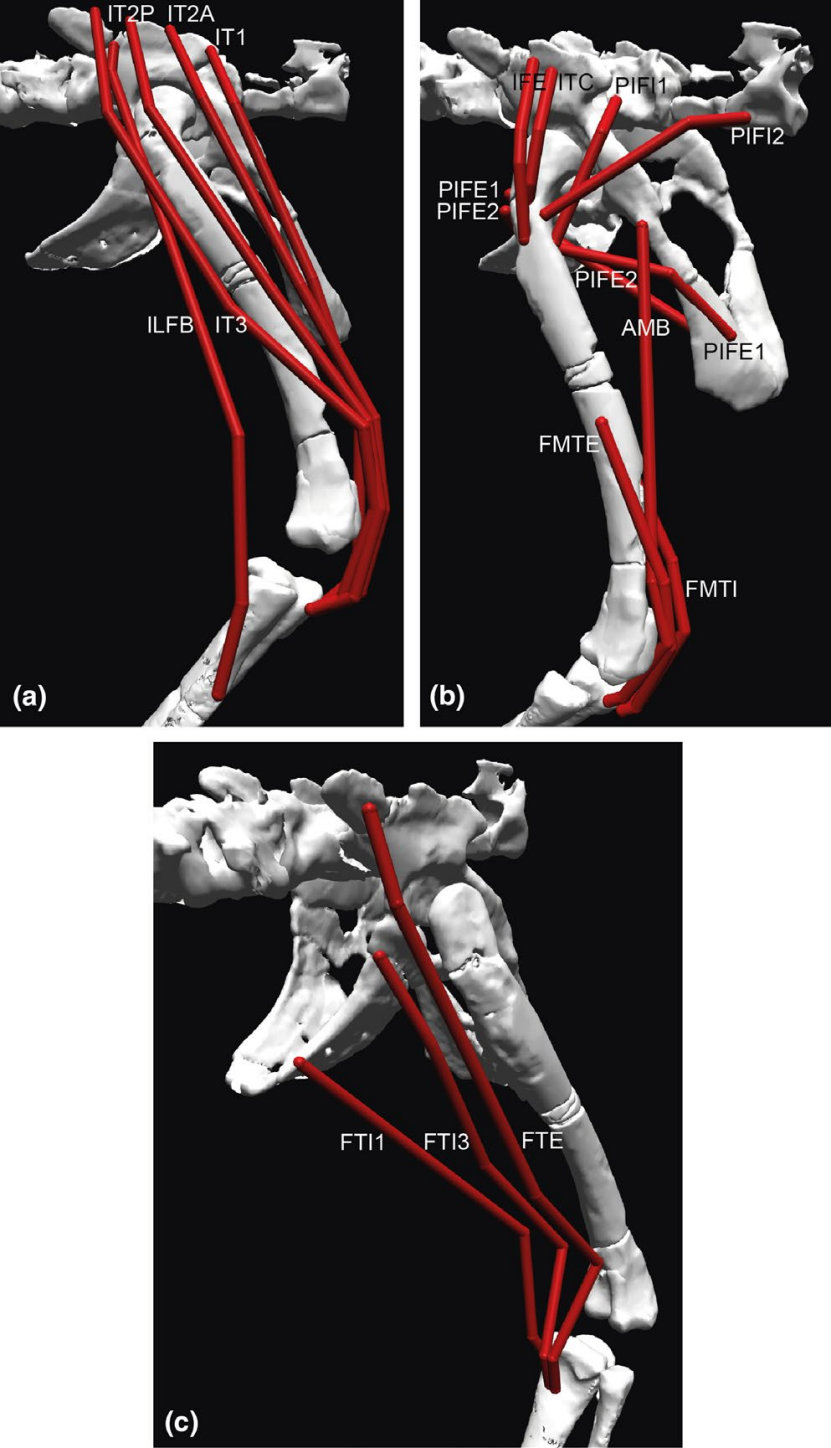
Muscles of the right pelvic limb implemented in the 3D musculoskeletal model; abbreviations are in Table 4. Superficial thigh muscles in lateral view (a), deeper thigh muscles in craniolateral view (b), and ‘flexor cruris’ hip extensor/knee flexor muscles of the thigh in caudolateral view (c). The model is in the limb pose used for estimating maximal muscle moments.

**FIGURE 5:**
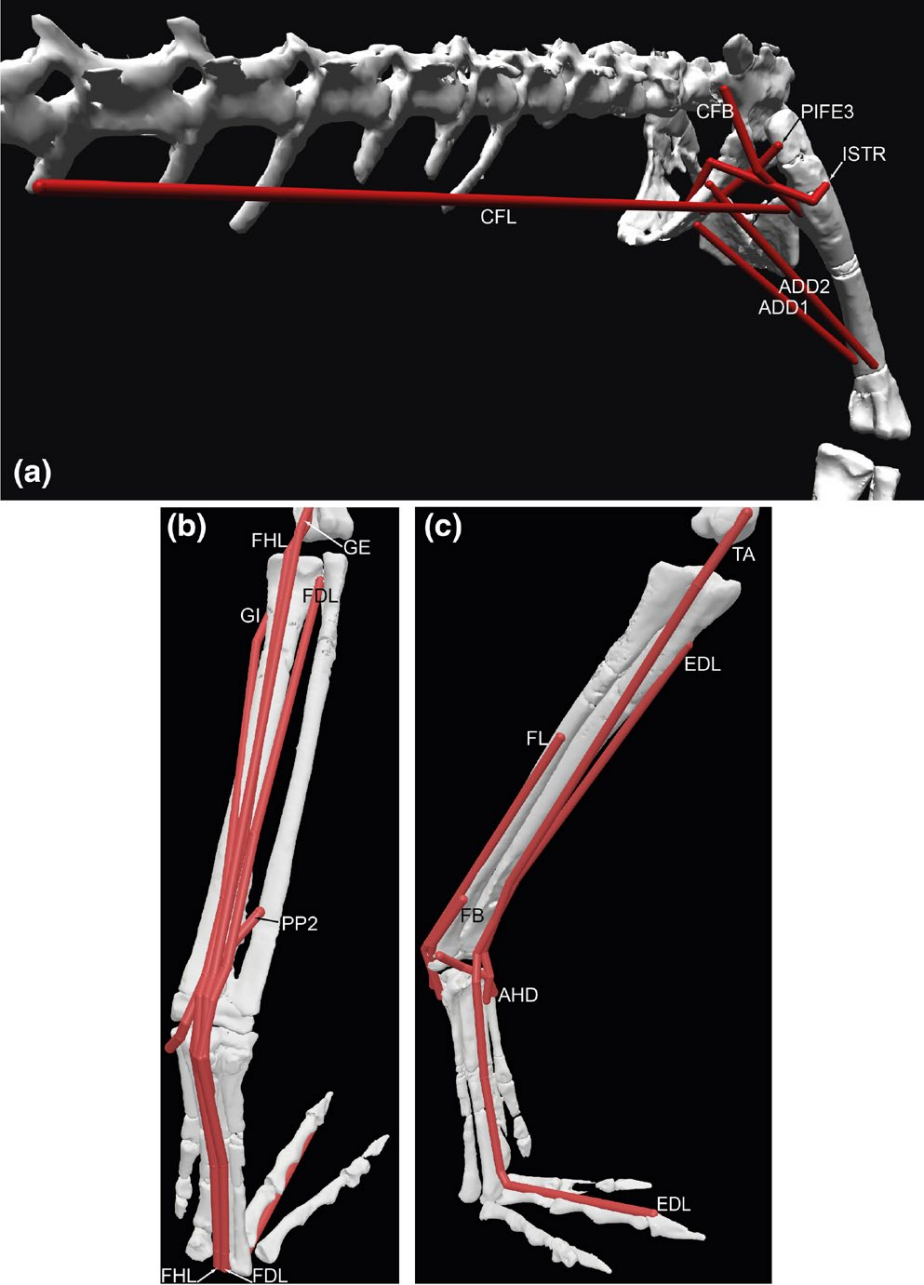
Muscles of the right pelvic limb implemented in the 3D musculoskeletal model; abbreviations are in Table 4. Deep thigh muscles in caudolateral view (a), Ankle extensor/digit plantarflexor muscles in caudolateral view (b), and ankle flexor/digit dorsiflexor muscles in craniolateral view (c). The model is in the limb pose used for estimating maximal muscle moments.

For Objective 2 (related to Aim 4; constructing a 3D musculoskeletal model), we added hindlimb musculotendinous paths around the skeletal coordinate system, adding ‘via points’ and ‘wrapping surfaces’ to constrain the paths appropriately, following Allen et al. (2021); Bishop, Cuff, and Hutchinson (2021); Hutchinson et al. (2005, 2015); Otero et al. (2017). Those paths' origins and insertions were based on a myological reconstruction using the extant phylogenetic bracket (Witmer, 1995) and a data matrix (in Mesquite v3.81 software; Maddison & Maddison, 2023), updated from Hutchinson (2001a, 2001b, 2002) and Bishop, Cuff, and Hutchinson (2021) (matrix and further details are in the Supplementary Information). Inferred muscle attachments are in Table 4; distal limb muscle homologies followed Hattori and Tsuihiji (2020); additional consideration of recent papers by Pereyra et al. (2023) and Wilhite (2023) was added. These muscles are detailed in Figures 4 and 5.

**TABLE 4:** Pelvic appendage muscle names, abbreviations, origins, insertions and levels of inference for *Lagosuchus*. I‐III = levels of inference as per Witmer (1995); ´ = level of inference lacking a clear osteological correlate (just relative position inferred). Only those muscles used in the musculoskeletal model are listed (see Supplementary Information for details), and attachments often were simplified in that model because it uses lines of action rather than 3D volumes.

Muscle	Origin	Insertion
M. iliotibialis 1 [IT1]	Craniodorsal iliac rim (roughening) [I]	Cranial tip of cnemial crest of tibia [I]
M. iliotibialis 2 [IT2]	Mid‐dorsal iliac rim (roughening) [I"]	Cranial tip of cnemial crest of tibia [I]
M. iliotibialis 3 [IT3]	Caudodorsal iliac rim (roughening) [I]	Cranial tip of cnemial crest of tibia [I]
M. femorotibialis externus [FMTE]	Lateral femoral shaft between intermuscular lines [I]	Cnemial crest of tibia [I]
M. femorotibialis internus [FMTI]	Medial femoral shaft between intermuscular lines and other muscle scars [I]	Cnemial crest of tibia [I]
M. ambiens [AMB]	Pubic tubercle of proximal pubis [I]	Cnemial crest of tibia [I]; secondary tendon to digital flexor origin [I´]
M. iliofibularis [ILFB]	Lateral surface of postacetabular iliac fossa, between IF and FTE [I]	Iliofibular tubercle on craniolateral proximal fibular shaft [I]
M. iliotrochantericus caudalis [ITC]	Lateral surface of ilium above acetabulum, cranial to IFE[II]	Lesser trochanter on craniolateral proximal femur [I]
M. iliofemoralis externus [IFE]	Lateral surface of ilium above acetabulum, caudal to ITC[II]	Caudolateral proximal femur, scarred raised area (trochanteric shelf) [I]
M. pubo‐ischio‐femoralis internus 1 [PIFI1]	Medial ilium and proximodorsal puboischiadic plate [II]	Craniomedial proximal femoral shaft, lateral to fourth trochanter [I´]
M. pubo‐ischio‐femoralis internus 2 [PIFI2]	“Lumbar” (caudalmost dorsal) vertebrae close to preacetabular ilium; lateral central surfaces [II]	Craniolateral proximal femur, near lesser trochanter [I´]
M. flexor tibialis internus 1 (FTI1)	Lateral surface of distal ischial shaft [II´]	Medial proximal tibia [I´]
M. flexor tibialis internus 3 (FTI3)	Proximal ischial tuberosity [II´]	Caudal proximal tibia [I´]
M. flexor tibialis externus (FTE)	Lateral surface of caudoventral corner of postacetabular ilium, caudal to ILFB [I´]	Caudal proximal tibia [I´]
M. puboischiofemoralis externus 1 (PIFE1)	Cranial surface of pubic apron [I]	Greater trochanter [I]
M. puboischiofemoralis externus 2 (PIFE2)	Caudal surface of pubic apron [I]	Greater trochanter [I]
M. puboischiofemoralis externus 3 (PIFE3)	Lateral surface of ischial apron, caudodorsal to ADD1 [I]	Greater trochanter [I]
M. ischiotrochantericus (ISTR)	Medial surface of ischial apron [I]	Lateral side of proximal‐most femur near trochanteric shelf and PIFE1‐3 [I]
M. caudofemoralis brevis (CFB)	“Brevis” fossa of ilium, and proximal caudal vertebrae [I]	Caudolateral side of proximal fourth trochanter [I]
M. caudofemoralis longus (CFL)	Lateral surfaces of haemal arches/chevrons and transverse processes of proximal‐to‐middle caudal vertebrae [I]	Fourth trochanter of femur; pit [I]
M. adductor femoris 1 (ADD1)	Craniolateral surface of ischial apron and shaft [I´]	Caudomedial distal femoral shaft; scarring [I]
M. adductor femoris 2 (ADD2)	Caudolateral surface of dorsal ischial shaft, from scarred groove [I]	Caudolateral distal femoral shaft; scarring near caudal intermuscular line [I]
M. gastrocnemius internus (GI)	Medial side of cnemial crest of proximal tibia [I´]	Calcaneal tuber, caudal side of distal tarsals and metatarsal V [I]
M. gastrocnemius externus (GE)	Proximal to lateral femoral condyle [i´]	Calcaneal tuber, caudal side of distal tarsals and metatarsal V [I]
M. extensor digitorum longus (EDL)	Lateral side of the cnemial crest; distal to TA origin; and the cranial tibial shaft [II]	Dorsal surfaces of the distal pedal phalanges [I]
M. tibialis anterior (TA)	Craniolateral side of the distal femur, and lateral side of cnemial crest [II]	Craniomedial sides of proximal metatarsals II‐IV [II]
M. flexor digitorum longus (FDL)	Proximomedial fibula's shaft [I´]	Flexor tubercles of pedal unguals II‐IV [I]
M. flexor hallucis longus (FHL)	Caudolateral distal femur near ge origin, lateral side of cnemial crest of the tibia, fossa flexoria, and proximal fibula [II′]	Flexor tubercles of pedal unguals I‐IV [I]
M. fibularis longus (FL)	Lateral shaft of fibula, distal to ILFB insertion [I´]	Caudal side of metatarsal V; distal to FB [II]
M. fibularis brevis (FB)	Distalmost shaft of fibula, distal to FL origin [I´]	Caudal side of metatarsal V; proximal to FL [II]
M. pronator profundus (PP)	Caudomedial/lateral fibular/tibial shafts [II]	Caudolateral side of metatarsal I and the process of distal tarsal IV [II]
M. abductor hallucis dorsalis (AHD)	Craniolateral side of distal fibula [II]	Proximodorsal (cranial) surface of metatarsal I, near EDL insertion [II]

**FIGURE 6:**
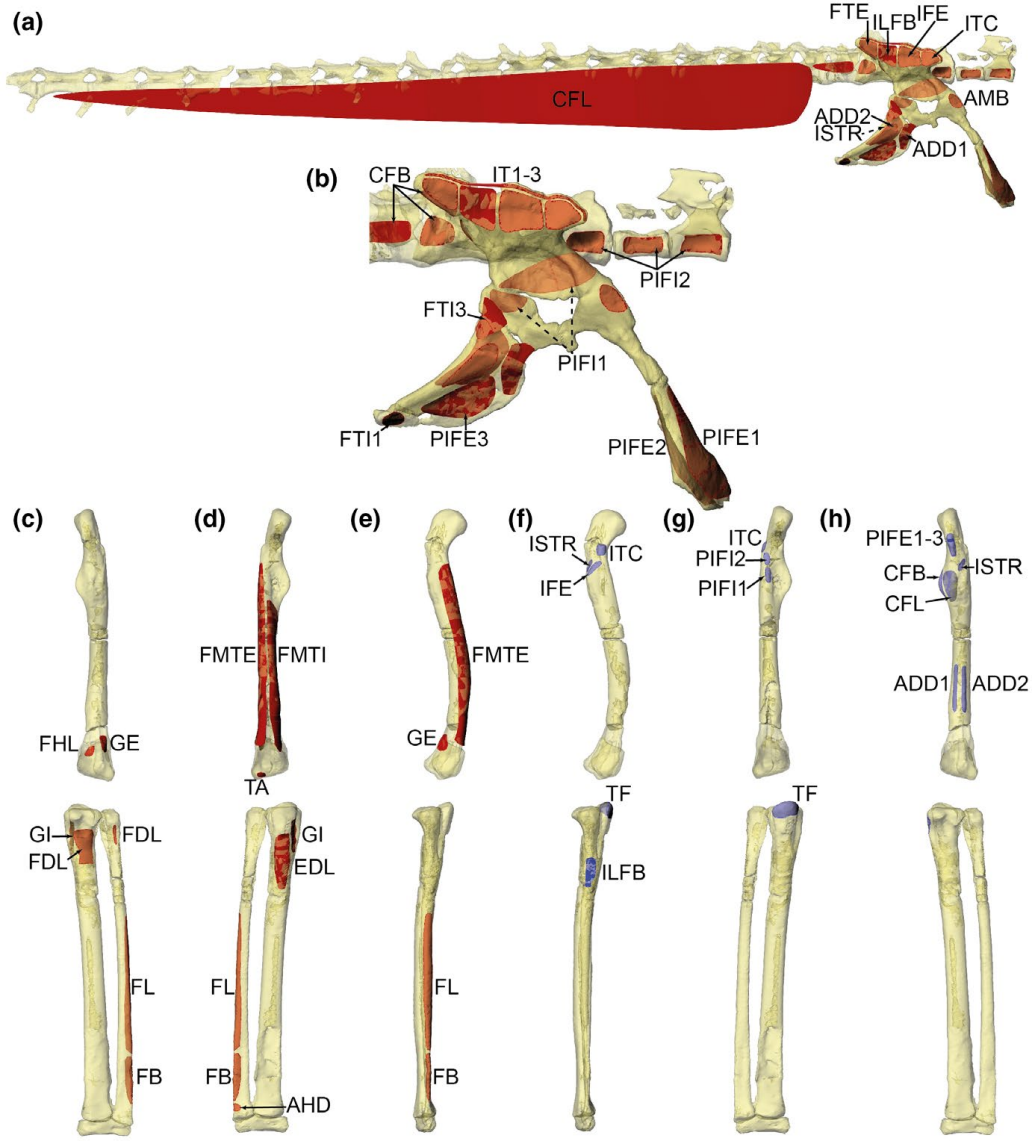
Muscle maps of origins (red) and insertions (blue) for quantifying AAs in the *Lagosuchus* model. Muscle abbreviations (and origin and insertion details) follow Table 4. Origins of muscles from the pelvis and caudal vertebrae in right lateral view (a) and from the pelvis (“zoomed in”) in right lateral view (b); and muscle origins from the femur and tibiotarsus in caudal (c), cranial (d) and lateral (e) views, and muscle insertions onto the femur and tibiotarsus in lateral (f), cranial (g) and caudal (h) views. Dotted lines for ISTR and PIFI1 origins indicate medial positions; ISTR is behind ADD2 and larger than it. Not to scale.

For Objective 3 (related to Aim 5), we used two main methods to estimate each muscle's maximal isometric force‐generating capacity (F_max_; via physiological cross‐sectional area; PCSA) in *Lagosuchus*. First (here “Method 1”), we used the attachment area (AA) method from Cuff et al., 2023 in Rhinoceros, corresponding to a ‘muscle map’ (Figure 6) of origins and insertions for the major hindlimb muscles with which we selected vertices on the bone meshes around the perimeter of the AAs and then calculated the total osteological area enclosed. As per Cuff et al. (2023), we computed the CFL muscle insertion AA, but we also computed the AA for the CFL origin, using the entire hypaxial space between vertebrae (Figure 6). The caudomedial surface of the fibular shaft was too poorly preserved to reconstruct a reliable AA for the PP muscle, and comparative data are extremely limited, so we omitted that muscle. We also did not reconstruct AAs for the small tendons of insertion of the muscles that insert on the pes. For comparisons, we used the same ‘muscle map’ AA model of the theropod dinosaur *Coelophysis bauri* from Cuff et al. (2023): their Table 7), including the CFL origin's AA. We converted these AAs into estimated PCSAs using the equations in (Cuff et al. (2023): their Table 5). AAs for extant archosaurs (*Crocodylus niloticus* [5 specimens; averaged] and *Eudromia elegans* [6 specimens; averaged]) are data taken from Cuff et al. (2023), which involved dissection and digitisation.

**TABLE 5:** Comparative body segment parameters (BSPs) of *Lagosuchus* model with models of *“Marasuchus”* (from Allen et al., 2013; mean model; Figure S1) and *Coelophysis* (from Bishop, Cuff, & Hutchinson, 2021). COM_x_ = dimensionless COM position along craniocaudal (for axial segments) or proximodistal (for appendicular segments) axis, for most models; non‐pedal digit COMs for *Coelophysis* are along the y‐axis, not x‐axis. We omit head and neck COMs and forelimb COMs because the models were designed too differently to meaningfully compare these. All hindlimb segment COMs are absolute values because coordinate systems varied. Pedal digit COMs are along the y‐axis for *Lagosuchus*; x‐axis for *Coelophysis*. Segment lengths used to non‐dimensionalise COMs are between JCSs. Hindlimb COM_x_ values for *Lagosuchus* and *Coelophysis* exclude the digits. The *“Marasuchus”* model was constructed differently, so not all segments were subdivided for comparison, or could have COM_x_ values presented.

Model	*Lagosuchus talampayensis*		*“Marasuchus lilloensis”*		*Coelophysis bauri*	
BSP	Mass/Body mass	COM_x_/Length	Mass/Body mass	COM_x_/Length	Mass/Body mass	COM_x_/Length
Proximal tail	0.265	−0.386			0.220	−0.332
Distal tail	0.041	−0.342			0.0164	−0.424
Whole tail	**0.306**	**−0.193**	**0.176**	**−0.328**	**0.236**	**−0.178**
Body	0.080	−0.144			0.175	0.116
Trunk	0.258	0.452			0.274	0.407
Body and trunk	**0.338**	**0.301**	**0.457**	**0.487**	**0.449**	**0.211**
Head and neck	**0.133**		**0.0624**		**0.128**	
Forelimb	**0.018**		**0.0334**		**0.0047**	
Thigh	0.039	0.405	0.0492		0.0501	0.547
Crus	0.042	0.537	0.0492		0.0309	0.521
Tarsometatarsus	0.0086	0.448	0.0169		0.0067	0.400
Pedal digits	0.0036	0.296	0.0039		0.0012	0.210
Hindlimb	**0.0938**	**0.069**	**0.119**	**0.0149**	**0.0890**	**0.181**
Whole body	**[0.134 kg]**	**0.0059**	**[0.285 kg]**	**0.0611**	**[13.1 kg]**	**0.0418**

*Note:* Bold entries emphasise data for combined body segments.

Second (here “Method 2”), we used the approach of Sellers et al. (2009, 2017; as used by Bishop, Cuff, & Hutchinson, 2021; see also Bishop, Michel, et al., 2021), which involved estimating muscle volumes from proportions in three cursorial mammals (here setting one hindlimb's total muscle mass as 11.1% body mass), then calculating PCSAs from volumes divided by muscle fibre (assumed equal to fascicle) lengths. Those fibre lengths were computed from the maximal minus minimal muscle‐tendon unit length changes across the maximal flexion/extension ROMs of the limb joints crossed by those muscles; for multi‐articular muscles, the greatest total length change across those joints' ROMs was used. Note that Sellers et al. (2013) followed a different approach for calculating fibre lengths, in which length change times two was used. Muscle architecture then was input into our musculoskeletal model following the conventional formula: maximal isometric muscle force F_max_ = PCSA × 0.3 N mm^−2^ maximal isometric stress (as per Medler, 2002; Michel et al. 2021 and references therein). Muscle pennation angle (an independent parameter in the OpenSim models) was irrelevant in these simple models and so was not addressed (see Lieber, 2022).

For comparisons with other taxa, where relevant we converted data from models to dimensionless form assuming isometry; for example, dividing linear dimensions by hindlimb length or body mass^0.33^, areas by body mass^0.67^. First, we compared estimated AAs from Method 1 between *Lagosuchus*, *Coelophysis* and two extant archosaurs (Nile crocodile *Crocodylus niloticus* and Elegant‐crested tinamou *Eudromia elegans*; Cuff et al., 2023) to explore how closely these muscle AAs follow general trends in the musculoskeletal evolution of the archosaurian pelvic limb (e.g., Hutchinson, 2001a, 2001b; Rhodes et al., 2021). Next, we compared with Bishop, Cuff, and Hutchinson's (2021) model of *Coelophysis* in detail, including dimensionless maximal isometric muscle moment‐generating capacities. We focussed on ‘antigravity’ muscles: hip, knee and ankle extensors and MTP plantarflexors. To obtain necessary data, we posed the *Lagosuchus* model in a static limb orientation (Figure 2b) similar to one previously used for *Coelophysis* (“Posture 2″ from Bishop, Cuff, & Hutchinson, 2021), and which is easily permissible by joint ROMs; justifiable as their *in vivo* limb orientations are unknown but presumed to have been similarly upright, unlike the more crouched poses in extant birds. Differences in limb pose were necessary to place the hindlimb in a more upright pose required to position the relatively much more caudal COM position in *Lagosuchus* (detailed in the “Body shape and segment dimensions of the 3D model” section of the Results and Discussion) over the middle of the digits and to keep the digits flat upon the substrate (*Lagosuchus* flexion/extension angles of right hindlimb = hip −20°, knee 55°, ankle −45°, MTP −10°; *Coelophysis* = hip −45°, knee 70°, ankle −44.5°, MTP −20.5°). We used the Analysis tool in OpenSim to quantify muscle moment arms in that pose; and calculated maximal muscle moments using these and F_max_ values from Methods 2 above (intrinsic force–length relationships were ignored here).

For comparisons with *Coelophysis*, we generally focus here on averaged moment arms for particular joints; this must be recognised as a simplistic first‐pass assessment, because muscle PCSAs will heavily influence moment‐generating capacities and thus may produce patterns that depart from those illustrated by average moment arms. Until joint JCSs, ROMs or habitual limb poses and muscle PCSAs (i.e., F_max_) can be more reliably estimated for extinct taxa, we see this as an acceptable, preliminary assessment of some basic questions about comparative biomechanics.

## 3 RESULTS AND DISCUSSION

Here we outline our main results and their broader implications, and comparisons with the literature; following our five main aims, to: describe osteological details from scanned specimens; construct a composite model; reconstruct body dimensions; build a musculoskeletal model including estimates of muscle architecture; and use the 3D model to estimate muscle moment‐generating capacities.

### 3.1 Observations on osteology

First, as per Table 1, to achieve our Aim 1, we studied six main PVL specimens catalogued as *Lagosuchus* (*talampayensis*); these were also investigated by Agnolín and Ezcurra (2019); Fechner (2009); Romer (1971, 1972) and Sereno and Arcucci (1994). As this is not meant to be a complete description, we only focus on noting differences among specimens, or novel observations of a specimen, that we consider important and not fully addressed in previous contributions.

#### 3.1.1 PVL 3870

PVL 3870 consists of a well‐preserved pelvis and sacrum, most of the left (and partial right) hindlimbs, cervical vertebrae 1–9, dorsal vertebrae ~10–19, and caudal vertebrae 1 + 2, as well as 13 mid‐caudals, plus part of the braincase and left maxilla. These have been well‐described by the references cited above so we will not elaborate further. However, Fechner (2009) noted that this specimen presents marked differences with PVL 3871 (see below), and thus considered it as a non‐dinosauriform dinosauromorph different from *Lagosuchus*. Agnolín and Ezcurra (2019), however, noted that the PULR 09 holotype's preserved pelvic girdle morphology (as much as is visible through the surrounding matrix) matches that of PVL 3870 well. Understanding PVL 3870's morphology depends on the question of what PVL 3871 is, as follows.

#### 3.1.2 PVL 3871 summary

PVL 3871 includes a left forelimb; composed mostly of a complete scapulocoracoid, humerus, radius and ulna; and right and left tibiotarsi, fragmentary pedal phalanges, a partial pelvis, and about 33 caudal vertebrae. In addition, this specimen also includes a right femur articulated with the acetabulum area of the right ilium (Romer, 1972; Sereno & Arcucci, 1994, Figure 8). Such material was included in the PVL 4670 specimen box (see comments below); however, that material has a red dot on it (indicating a holotype), as PVL 3871 does; hence we attribute the right femur with a portion of the acetabulum to PVL 3871, in agreement with prior studies (e.g., Sereno & Arcucci, 1994). Although PVL 3871 has been sufficiently described (Agnolín & Ezcurra, 2019; Bonaparte, 1975; Romer, 1972; Sereno & Arcucci, 1994), some main differences with remaining materials assigned either to “*Marasuchus*” or *Lagosuchus* have not been properly addressed.

#### 3.1.3 PVL 3871 left scapulocoracoids

**FIGURE 7:**
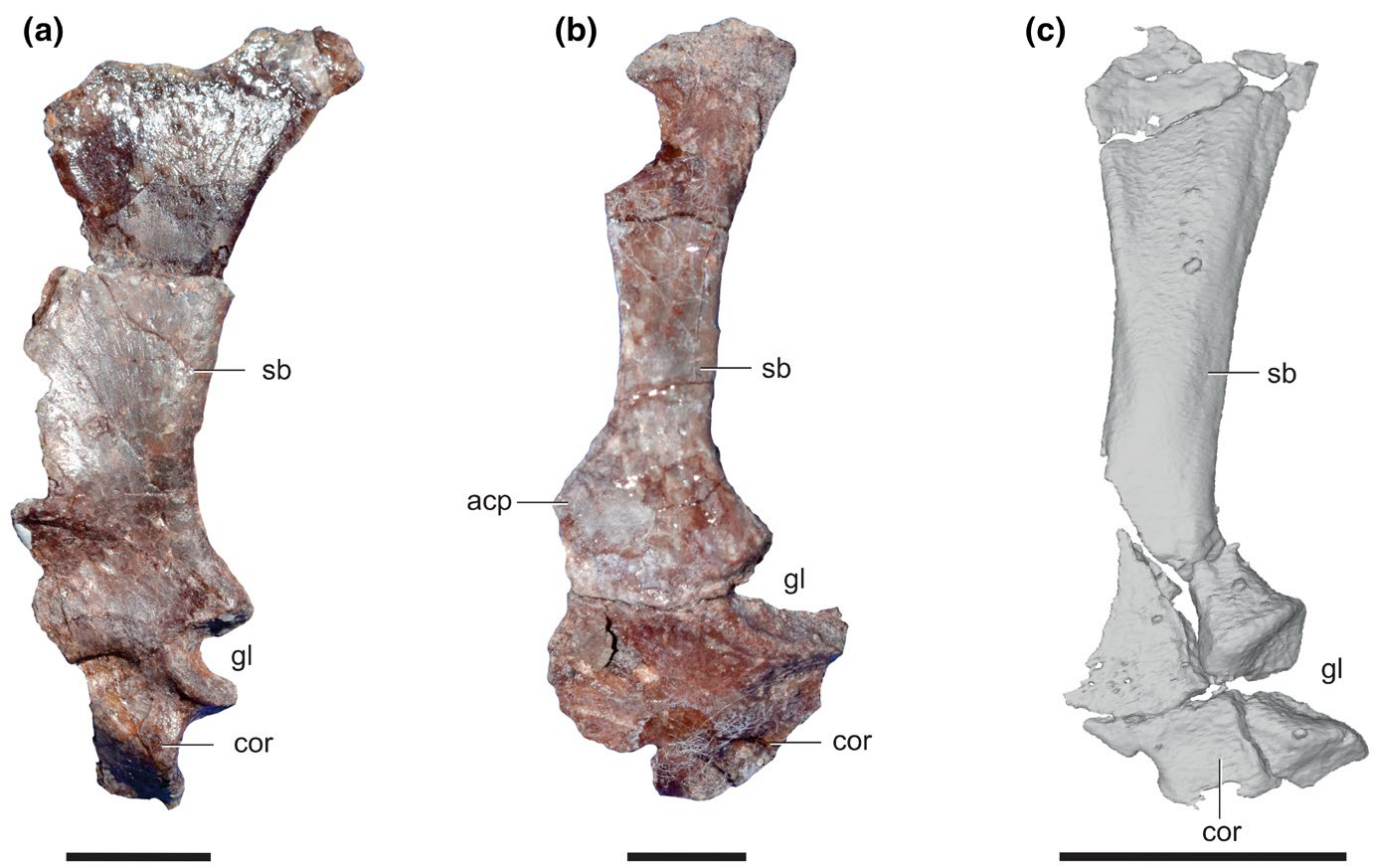
Scapulocoracoid of *Lagosuchus talampayensis*. (a), left scapulocoracoid (PVL 3871); (b), right scapulocoracoid reversed (PVL 4672); (c), left scapulocoracoid (PVL 3871). acp, acromion process; cor, coracoid; gl, glenoid; sb, scapular blade. Scale bar equals: 5 mm (a, b), 10 mm (c).

There is also an additional left scapulocoracoid embedded in the matrix in the same slab as the remaining bones of PVL 3871 (Figure 7c), which obviously creates a conflict with the actual assigned left scapulocoracoid (Figure 7a). The originally described left scapulocoracoid of PVL 3871 (Figure 7a; Bonaparte, 1975: their Figure 8; Remes, 2008: their Figures 4–7a) is quite different from the additional element embedded in the matrix (Figure 7c) and also from PVL 4672 (see below). The former left element from PVL 3871 is proportionally shorter (19 mm long) and has a markedly more expanded blade (4 mm wide at base). These attributes have repeatedly been noted (Bonaparte, 1975; Sereno & Arcucci, 1994, their p. 60; Remes, 2008, their p. 147), including similarities with the ‘sphenosuchians’ *Hesperosuchus* and *Pseudohesperosuchus*. We will not repeat the convincing case for this attribution; Remes (2008, their p. 147) summed up the situation well: “these elements are sphenosuchian in origin, and were accidentally added to the *Marasuchus* type material by Bonaparte (1975)”. However, the morphology of the additional left scapulocoracoid curated with the other PVL 3871 material is conspicuously divergent from that of the other left side element, appearing more elongate (29 mm) and relatively narrower (4 mm) at the base (Figure 7c). We are not aware of other studies describing this material, which became apparent in our scans. We consider the issue of scapulocoracoid identification more thoroughly below with PVL 4672.

**FIGURE 8:**
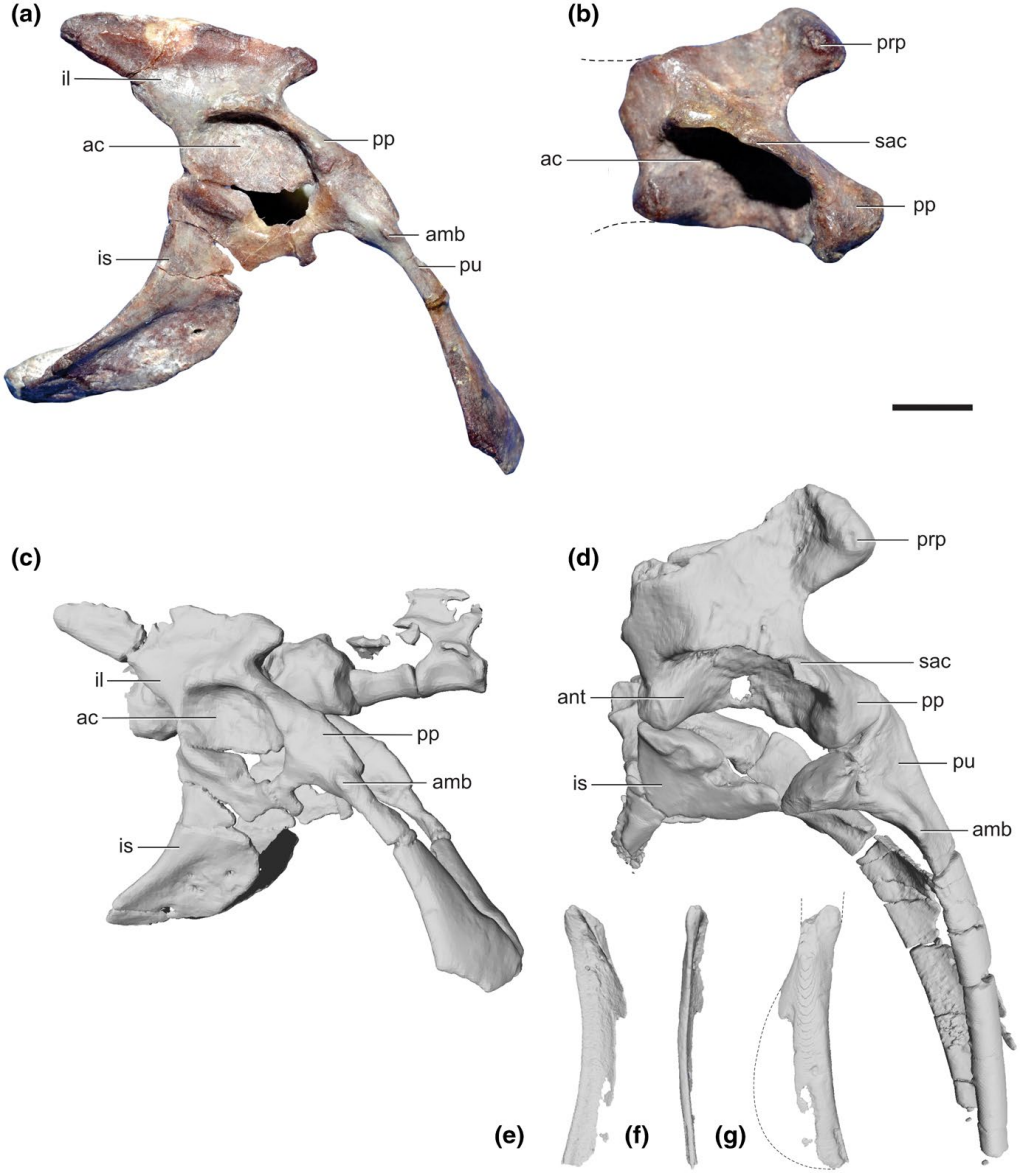
Pelvis of *Lagosuchus talampayensis* and putative silesaurid pelvis. Complete right pelvis of *L. talampayensis* (PVL 3870) in lateral view (photo in (a), 3D reconstruction in). Pelvic elements of silesaurid(?) (PVL 3871; photo in (b), 3D reconstruction in (d), flipped from left). Incomplete left pubis of PVL 3781 in caudal (e), lateral (f) and cranial (g) views. ac, acetabulum; amb, ambiens process; ant, antitrochanter; il, ilium; is, ischium; pp., pubic peduncle; prp, preacetabular process; pu, pubis; sac, supra‐acetabular crest. Scale bar equals 5 mm.

#### 3.1.4 PVL 3871 ilium

The ilium of PVL 3871 (Figure 8b,d), as preserved, presents notable differences from PVL 3870 (Figure 8a,c). The preacetabular lobe of the left ilium is pointed in PVL 3870 (quite similar to the lagerpetid *Ixalerpeton*, Cabreira et al., 2016) but more square‐shaped in PVL 3871. Furthermore, the cranial outline formed by the preacetabular lobe and the pubic peduncle forms a triangular shape in PVL 3870, being sub‐circular in PVL 3871. Moreover, there is a distinct supra‐acetabular crest in the latter, not observed in the former. The pubis also has noteworthy differences between both specimens, being substantially shorter in PVL 3870 and lacking the large, slightly distally placed ‘ambiens’ (AMB origin) process present in PVL 3871 (left pubis; 30 mm preserved length). Importantly, the shape of the preacetabular lobe, the angle of the latter and the pubic peduncle, the well‐developed supra‐acetabular crest and the length of the pubic apron observed in PVL 3871 are a combination of diagnostic traits shared with *Silesaurus* (Figure 11 in Dzik, 2003; also see Piechowski and Tałanda, 2020), and other known silesaurids with pelvic material (Langer et al., 2013; Martz & Small, 2019; Nesbitt et al., 2010, 2020; Peecook et al., 2013; Pretto et al., 2022; Sullivan & Lucas, 1999); and not other known archosaurs. Although Sereno and Arcucci (1994, Figure 6a,b) reconstructed the PVL 3870 ilium with the same shape of PVL 3871, the above‐mentioned differences between both pelves are not considered here as a result of poor preservation.

#### 3.1.5 PVL 3871 pubes

Curiously, there is another hitherto unnoticed element in the box with this PVL 3871 material, and its morphology (including size) is almost a precise match to that of the left pubis (distal to the ambiens process, continuing to the complete distal end, 25 mm in preserved length; Figure 8e–g). Hence there are two left pubes curated together; possibly from the same taxon. Agnolín and Ezcurra (2019) did not discuss either. Romer did not aid clarity by cryptically commenting that, for PVL 3871, “Parts of pubis and ischium are preserved in this specimen… in default of a better preserved specimen I refrain from discussion of this portion of the girdle.” (Romer, 1971, their pp.7–8); and (Romer, 1972, their p.7) “Much of the pelvis is preserved. Interpretation of its peculiar structure should best be delayed until a more complete specimen is found”. Assuming that Romer (1971) is referring to PVL 3871 and lacking any other field/museum notes, we only can tentatively conclude that an additional specimen of *L. talampayensis* was present and included together with PVL 3871 during material curation.

#### 3.1.6 PVL 3871 pelvis comparative morphology

*Lewisuchus admixtus* (e.g., holotype of “*Pseudolagosuchus*”, PVL 4629; also PVL 3454) is the only currently known silesaurid from Argentina (Agnolín et al., 2021; Arcucci, 1987; Bittencourt et al., 2015; Ezcurra, Nesbitt, Fiorelli, & Desojo, 2020; Romer, 1972), and comes from the same geologic formation as the pelvic elements of PVL 3871. Although the partially preserved ilium of PVL 3871 (Figure 8b,d; Sereno & Arcucci, 1994: their Figure 5a) is similar to new *Lewisuchus* material (Ezcurra, Nesbitt, Fiorelli, & Desojo, 2020: their figure 22; Agnolín et al., 2021: their Figure 8), the preacetabular process of PVL 3871 presents a ‘bump’ placed cranioventrally, producing a rounded cranial contour of the process. In *Lewisuchus* material (PULR V‐111, Agnolín et al., 2021), such a bump is not present, producing a more squared cranial shape of the preacetabular process. PVL 3871 lacks the rodlike pubic shafts (they are broader, with a substantial “apron”; Figure 8d–g) listed by Ezcurra, Nesbitt, Fiorelli, and Desojo (2020) and Agnolín et al. (2021) as diagnostic for *L. admixtus*. The brevis fossa region of the ilium is unpreserved, so their second diagnostic trait “ilium with a shallow and ventrally facing brevis fossa” cannot be compared here. Yet based on the pubic morphology, the pelvis does not seem to pertain to *L. admixtus*, and thus it putatively represents a second silesaurid taxon from Argentina. Future studies can determine if it has autapomorphies, or association with other material, that justify erecting a new taxon or not. We do not consider here if any other elements curated with PVL 3871 are silesaurids. Fechner (2009: their p. 19) noted that PVL 3871 differs from the other fairly well‐preserved specimen PVL 3870, suggesting that the latter specimen pertained to a new “non‐dinosauriform dinosauromorph” taxon, but this suggestion has not been explored further and they did not provide further details (except that it may have had a shorter trunk). Instead, we suggest that the PVL 3871 pelvis described here is a silesaurid taxon and PVL 3870 is best attributable to *Lagosuchus talampayensis*. Indeed, Fechner (2009: their Figure 4) depicted the PVL 3871 pelvis that we discuss here, but did not consider if it was a silesaurid.

#### 3.1.7 PVL 3871 problems summary

A key problem for assigning the right side of the pelvis and the left and right scapulocoracoids of PVL 3871, and the forelimb material of PVL 4670 and 4672 (see below), to a particular specimen or taxon/taxa is that no field notes or other metadata clearly are associated with any of these bones. Somehow these bones currently are in drawers with other material, and not catalogued as any other taxon or specimen number, but some are not described in the literature on *Lagosuchus*. Hence it presently is not possible to ascertain which bones, other than the main articulated ones of PVL 3871 on its slab, definitely are of *Lagosuchus “lilloensis”*.

#### 3.1.8 PVL 3871 articulated femur

Regarding the PVL 3871 right femur attached to the acetabulum, it shows strong correspondence (within the limits of preservation) to the morphology of the right and left femora of PVL 3870, in terms of the femoral head and neck (Figures 8 and 9 in Sereno & Arcucci, 1994), fourth trochanter, trochanteric shelf, muscle scars, shaft curvature, and distal femur. Thus we do not discuss any of these femora further (for details see Agnolín & Ezcurra, 2019; Bonaparte, 1975; Sereno & Arcucci, 1994).

**FIGURE 9:**
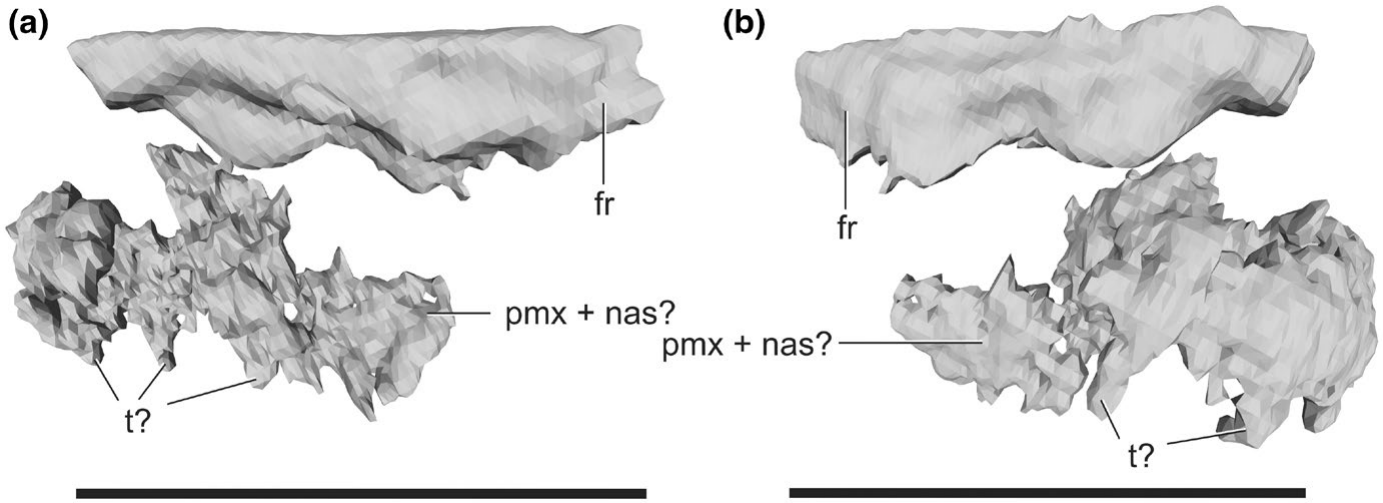
Rostral (?) remains of *Lagosuchus talampayensis* (PVL 3871). fr, frontal; nas, possible nasal; pmx, possible premaxilla; t, possible tooth. Scale bar equals 5 mm.

#### 3.1.9 PVL 3871 skull bones

From the scanned materials of PVL 3871, there is also a set of putative bones with similarities to rostral elements, although the preservation and image contrast in the CT scans make proper identification difficult (Figure 9). One of these elements (about 4.7 mm long, 1.8 mm tall and 2 mm wide) could be a fragment of premaxilla plus a nasal, in which possible tooth elements can be recognized rising from the former. Just above it in the scan, but not connected to it, there is another element (about 5.4 mm long, 1.4 mm tall and 1.1 mm wide) that could represent a frontal, considering its elongated proportions and the presence of a straight margin that could be the contact margin with its counterpart over the skull midline. We did not include these possible elements in our skull reconstruction because their identification is so tentative, and they are not closely associated with the other skull bones.

#### 3.1.10 PVL 3872 cranial material

**FIGURE 10:**
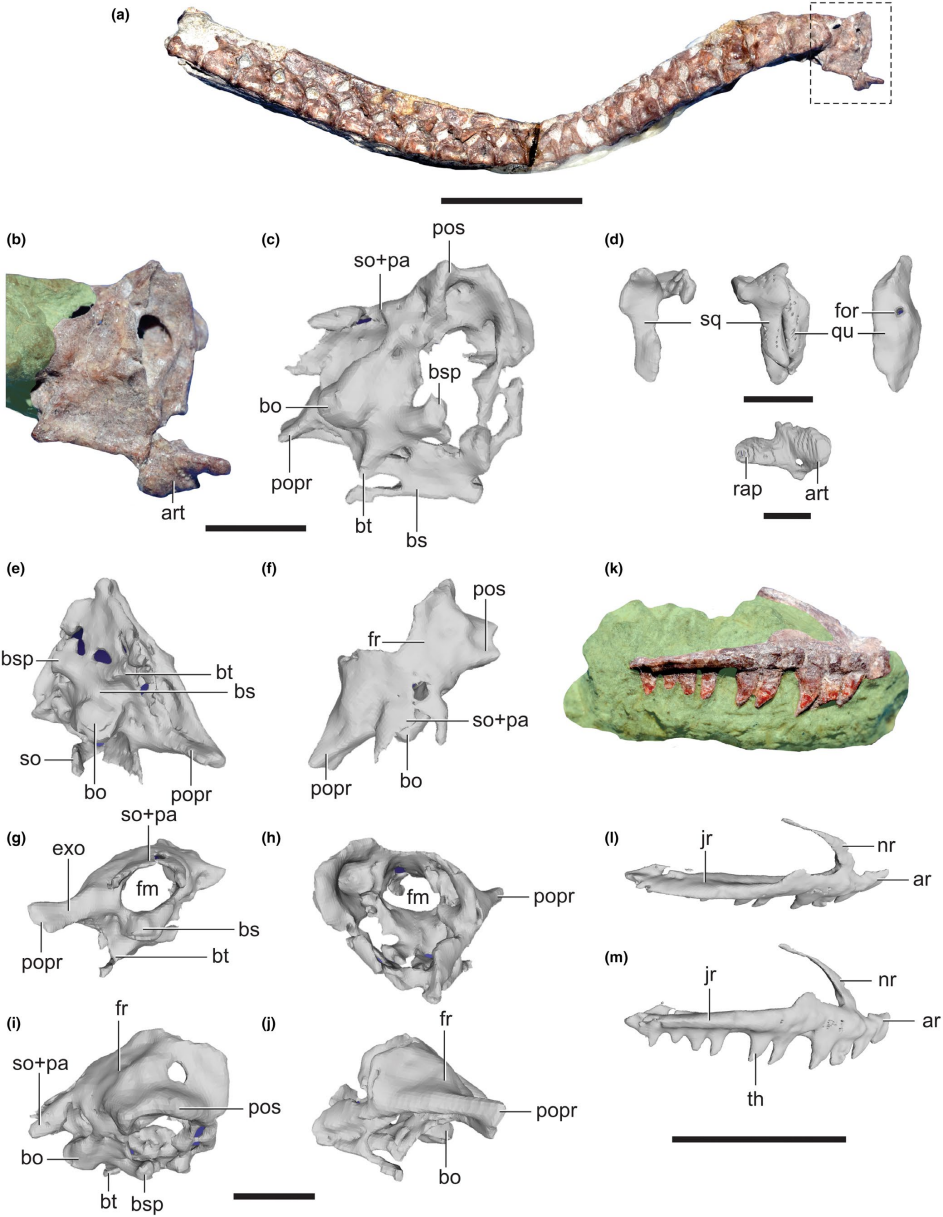
Cranial elements of *Lagosuchus talampayensis*. Skull elements of PVL 3872, showing basicranium attached to an articulated series of cervical vertebrae (a); right ventrolateral view of basicranium in a cropped photo (b) and 3D reconstruction (c); isolated cranial bones (d); 3D reconstruction of isolated basicranium and additional skull bones in ventral (e), dorsal (f), caudal (g), cranial (h), right lateral (I) and left lateral (j) views. Left maxilla of PVL 3870 in original matrix (k) and 3D reconstructions (l, dorsolateral; m, lateral). art, articular; bo, basioccipital; bs, basisphenoid; bsp, basisphenoid process; bt, basal tubera; exo, exoccipital; fm, foramen magnum; for, foramen; fr, frontal; jr, jugal ramus; nr, nasal ramus; pa, parietal; popr, paroccipital process; pos, postorbital; qu, quadrate; rap, retroarticular process; so, supraoccipital; sq., squamosal. Scale bar equals: 10 mm (a); 5 mm (B, K); 2 mm (d); 4 mm (e–j).

PVL 3872 has a braincase and jaw bones, cervical vertebrae 1–9, and dorsal vertebrae 1–13 (Figure 10a–j). We used that cranial material, plus the left maxilla and missing parts of the braincase from PVL 3870, to reconstruct the approximate shape of the skull, then scaled the sculpted NHMUK PV R14101 specimen to match this. We do not describe the braincase because prior studies have done so in detail (Bonaparte, 1975; Sereno & Arcucci, 1994). However, as we isolated and manipulated the 3D model of the skull remains, we were able to study the basicranium in different views, allowing us to ascertain the identity and relationships of several preserved bones, which are difficult to determine with the skull attached to the cervical series (Figure 10).

In caudal and ventral views (Figure 10e,g), the presence of the left paroccipital process is evident. The presence of the exoccipital bone was mentioned by Bonaparte (1975) and Sereno and Arcucci (1994); nonetheless, the continuation of the exoccipital bone in a paroccipital process is only visible from caudal and ventral views of the basicranium, as we provide here. The paroccipital process figured by (Sereno & Arcucci, 1994: their Figure 2) actually corresponds to a broken area that the squamosal should be occupying. The occipital condyle (basioccipital) is also evident in ventral and caudal views (Figure 10e,g), above which is the foramen magnum. The identification of this foramen allowed us to verify the identity of the bones that surround it, such as the supraoccipital fused to the parietal (Figure 10c,f). In dorsal view, cranial to the latter bones, a wide surface can be seen that should be occupied by the frontal, and lateral to it, the postorbital (Figure 10c,f,i,j).

Sereno and Arcucci (1994) reinterpreted the quadrate as a partial postorbital; this is not followed here (see also Agnolín & Ezcurra, 2019). Our scans revealed that the left articular (rostrocaudally about 4.3 mm long) of PVL 3872 was preserved close to an *in situ* articulation (Figure 10b), in addition to the right quadrate and squamosal (Figure 3 in Bonaparte, 1975; dorsoventrally the latter is about 4.3 mm long and the former 5.5 mm long) (Figure 10d). Its general morphology is similar to that of *Asilisaurus* (Nesbitt et al., 2020) and other silesaurids. The glenoid of the articular has two fossae for articulation with the distal quadrate (unpreserved), separated by a mediolateral ridge, and a strong ridge dividing the glenoid from the retroarticular process, which is longer than the rostral portion of the articular. The retroarticular process is robust and tuber‐like, with a quadrangular shape and a shallow fossa on the dorsal side of its caudal end. A foramen penetrates mediolaterally through the ventral side of a large fossa medial to the glenoid.

#### 3.1.11 PVL 4670 forelimb

The situation of PVL 4670 is confusing regarding the allocation of the materials that compose it. Only Agnolín and Ezcurra (2019) and Sereno and Arcucci (1994) mentioned it, as including a series of articulated proximal caudal vertebrae lacking chevrons; Bonaparte (1975) and Romer (1971, 1972) and and other prior studies of *Lagosuchus* did not mention it. Nonetheless, when one of the authors of this contribution (AO) was reviewing PVL 4670 firsthand, other elements were in the specimen's box, including an incomplete forelimb (partial humerus articulated with a radius and ulna), additional vertebrae and a right femur with a portion of the acetabulum (i.e., partial pelvis in articulation with femur; assigned to PVL 3871 by Sereno & Arcucci, 1994 [their Figure 8] as we discussed above). It is unclear where the forelimb and vertebral elements came from; they lack specimen numbers attached to the bones.

**FIGURE 11:**
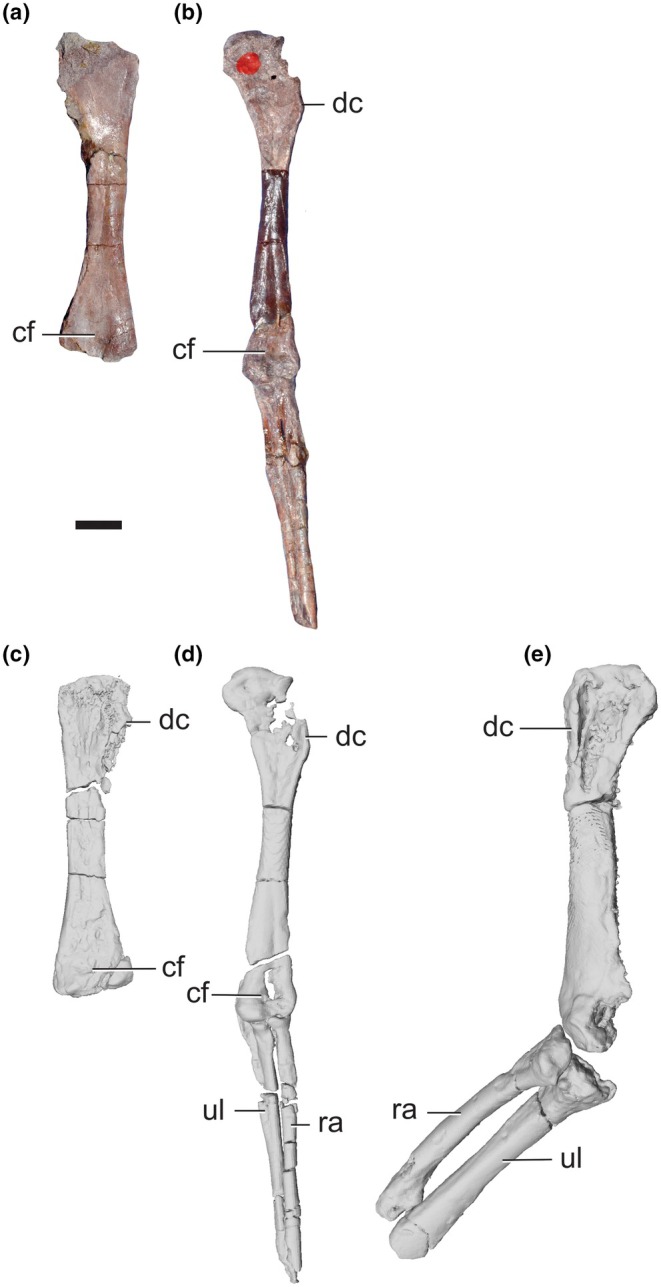
Forelimb elements of *Lagosuchus talampayensis*. Left humerus (PVL 4672) in cranial (a) and caudal (c) views; incomplete left forelimb (PVL 3871) in cranial view (b, d); incomplete right forelimb (PVL 4670) in cranial view (e). cf, cuboid fossa; dc, deltopectoral crest; ra, radius; ul, ulna. Scale bar equals 5 mm.

From the above material of PVL 4670, the forelimb is, perhaps, the most striking specimen because of its overall robustness, which markedly differs from that of PVL 3871 (Figure 11). In this regard, the minimum shaft transverse width / total humeral length of PVL 3871 is ~0.07 (2.8 and 38 mm), whereas in PVL 4670 it is ~0.11 (4.7 and 43 mm). The radius (34 mm long, 3.1 mm midshaft width) and ulna (33 mm long, 3.7 mm midshaft width) curated with PVL 4670 have the same robust morphology as the humerus compared with PVL 3871, albeit the bones of PVL 4670 experienced distortion via crushing. Agnolín and Ezcurra (2019) noted that PVL 3871 has a substantially greater ratio of the lengths of radius or ulna vs. humerus (>72%) than PULR 09 (65%); PVL 4670 has a ratio (>80%) more similar to PVL 3871. Similarly, they noted that PULR 09's deltopectoral crest on the humerus is relatively shorter vs. that in PVL 3871; and we observe the same for PVL 4670 (35% of humerus length). Finally, the ulna of PVL 4670 lacks the well‐developed olecranon present in PVL 3871, which extends proximal to the humeroulnar articulation. The holotype PULR 09 likewise lacks the well‐developed olecranon (Agnolín & Ezcurra, 2019). We thus conclude that, at least, the humerus, radius and ulna of PULR 09 and PVL 4670 are not from the same taxon as PVL 3871 (which might be a ‘sphenosuchian’ forelimb; see scapulocoracoid discussion above; and Remes, 2008).

#### 3.1.12 PVL 4671 and 4672

PVL 4671 simply has 23 proximal caudal vertebrae of a large specimen, previously described by Sereno and Arcucci (1994). PVL 4672 is composed of presacral vertebrae (including atlas) until the 17th presacral, previously described by Sereno and Arcucci (1994) and Agnolín and Ezcurra (2019). This material's box also includes a right scapulocoracoid and a left humerus (Agnolín & Ezcurra, 2019; Remes, 2008) but not mentioned by Sereno & Arcucci, 1994). Overall, the scapulocoracoid of PVL 4672 resembles that of early sauropodomorphs in being proximally and distally expanded and relatively short proximodistally (see also Remes, 2008). PVL 4672 has an acromial process on the cranial base of the scapula that rises from the scapular dorsal margin at an angle of about 120°, similar to the condition in *Silesaurus* and *Lewisuchus* (Dzik, 2003; Bittencourt et al. 2014), but differing from early dinosaurs like *Herrerasaurus* and *Eoraptor*, which have a more acute angle (Sereno, 1994; Sereno et al., 2012). That region is not preserved in PVL 3871 (either left scapulocoracoid). PVL 4672 is comparatively more slender than PVL 3871, with a less expanded scapular blade (33 mm long total; 4.5 mm wide at base), both at the base and distal end, relative to the left element in PVL 3871. The scapulocoracoids of PVL 3871 (Figure 7b) and 4672 (Figure 7c) share the cranially (= dorsally) curved, caudally (= ventrally) straight borders of the scapular shaft with other ornithodirans such as *Lewisuchus* and *Silesaurus* (Dzik, 2003; Remes, 2008); unlike the scapular shafts of “sphenosuchians” and the other PVL 3871 scapulocoracoid (Figure 7a), which are curved on both borders (Remes, 2008: their Figure 4,7a). Additionally, the glenoid outline is not circular as in PVL 3871's crocodylomorph‐like left element, and the coracoid lip of the glenoid is far more extended in PVL 4672 than in the former specimen. The left humeral shaft (37 mm length, 4.3 mm midshaft width) and distal expansion of PVL 4672 are proportionally more expanded than in PVL 3871, but the deltopectoral crest is shorter (35% of humerus length). The overall robustness of PVL 4672 (humerus and scapula) leads us to consider these two bones as pertaining to a different taxon/taxa from PVL 3871 (left scapulocoracoids and right forelimb), supporting Remes's (2008) (pp. 135–136) suggestion (*contra* Agnolín & Ezcurra, 2019).

#### 3.1.13 Forelimb morphology summary

Thus, including PVL 4670, there is substantial pectoral appendage material (Figures 7 and 11) known from at least two other Late Triassic archosaurs (apparently one ‘sphenosuchian’ and at least one dinosauriform), in addition to *Lagosuchus* (PULR 09), which needs further study and clearer identification. The restudy of the holotype and other specimens by Agnolín and Ezcurra (2019) did not resolve this issue, but agreed that the scapular blades of PVL 3871 and PVL 4672 are conspicuously broader than in PULR 09. Finally, as Remes (2008, p. 135) noted, Romer (1972) introduced confusion by describing a left scapulocoracoid (MCZ 4121; now MCZ 9483; not studied here) as pertaining to *Lagerpeton*, but Romer's (1972) Figure 2 captioned it as *Lagosuchus talampayensis*; and regardless Romer claimed that those two specimens were found in a nodule with the holotype of *Lewisuchus admixtus* (see below). Agnolín and Ezcurra (2019) and Remes (2008) cogently argued that the left scapulocoracoid specimen MCZ 9483 that Romer (1972) referred to *Lagerpeton* (or *Lagosuchus*?) is from some dinosauriform other than *Lewisuchus*, so there is further ambiguity surrounding potential comparative material. Mancuso et al. (2014) mentioned that perhaps MCZ “9483R” is proterochampsid, not *Lagosuchus* (and listed PVL 3871 as such, too).

### 3.2 Implications of osteological findings for musculoskeletal modelling

Our reassessment of the available material raises the question of what can be unambiguously referred to *Lagosuchus talampayensis*, what belongs to a separate taxon, and what has an unclear taxonomic assignment. More importantly in the context of locomotor function, how representative is our composite model (see Table 2 for elements used) of *Lagosuchus talampayensis*, and how might any remaining uncertainties matter for our musculoskeletal modelling?

All of PVL 3870 used here (the basis of much of our model except for the pectoral limbs) articulates (or is apparently associated) well enough that we conclude it is safe to consider as one individual. Although the vertebral material used in our model has no clear apomorphies according to the most recent description (Agnolín & Ezcurra, 2019), studies agree that there are no anatomical or phylogenetic reasons to exclude them from PVL 3870 (Agnolín & Ezcurra, 2019; Sereno & Arcucci, 1994). Hence, we do not question the assignment of known vertebral material used in our model (parts of PVL 3870, 3871, 4670, 4671) to *L. talampayensis*.

The articulated/associated hindlimb, caudal vertebrae and other elements of PVL 3871 seem to belong to *L. talampayensis* (Agnolín & Ezcurra, 2019). However, we found intriguing evidence that the incomplete pelvis (not articulated/clearly associated with the other elements, in our view; Figure 8b), at least, of PVL 3871 may not conclusively be associated with the main slab, or at least with what all prior studies conceived as *L. talampayensis*. That pelvis is either a previously unknown silesaurid, or (perhaps not mutually exclusive) “silesaurid” diagnostic traits of the pelvis might be homoplastic in silesaurids vs. *L. talampayensis—*or synapomorphies for Dinosauriformes (reversed in Dinosauria?). We cannot resolve that difficult question here. Follow‐up studies should revisit it in detail, and test it with phylogenetic analyses.

We have noted that some pectoral limb material is, or might, not plausibly be considered as pertaining to *L. talampayensis* (Figures 7a and 11e). In particular, there is the lingering concern that the pectoral limb (without a manus) from PVL 3871 as used here might not even be from a dinosauriform or other avemetatarsalian archosaur (see discussion above). Other PVL pectoral limb material that has been described or curated as part of *L. talampayensis* is either ambiguous (PVL 4672) or clearly from some other small ornithodiran with remarkably more robust forelimbs (PVL 4670). In part, this is because the scapulocoracoid of the *L. talampayensis* PULR 09 is not well preserved, complicating comparisons with other material preserving diagnostic traits. The scapulocoracoid has minimal effects on the body shape of our model, and to complete its form we combined the scapulocoracoids of PVL 3871 (Figure 7b specimen) and PVL 4672 (Figures 2c and 7c), manually scaling the latter down to match the other specimen. We opted to use the remainder of PVL 3871's pectoral limb (Figures 2c and 11b,d), in lieu of a better alternative. Only further studies can resolve the quandary we have outlined here, but we expect that different (within reason) dimensions of these bones as modelled would not have a large effect on the key parameters of body mass and centre of mass as quantified and used here; or on more complex analyses such as predictive simulations (see Bishop, Falisse, et al., 2021). However, because most studies have relied on the assumption that the seemingly short forelimbs vs. elongate hindlimbs of *Lagosuchus* indicate bipedal locomotion (see Introduction), which we also assume that to be the case here, this is an important issue to revisit in future studies that might resolve which (if any) forelimb material can be attributed to the genus.

Our model includes the assumption that missing parts of the skull and manus used from the NHMUK PV R14101 cast/sculpture are sufficient for our purposes. See Figure S1 for the morphology of that specimen. Indeed, we see no feasible alternative, as these regions are unknown for *L. talampayensis*. Overall, we argue that our model is a reasonable approximation of what is generally considered to be *L. talampayensis*. Surely there are at least relatively minor errors in scaling, taphonomy, individual variation, or other factors, but we expect these to have fairly trivial impact on our model's key parameters (mainly its general 3D geometry) that would have important downstream consequences for our quantitative model and simulation outputs. Our study's model cannot have the level of accuracy to represent the exact morphology of the individuals PVL 3870 or 3871 and their palaeobiology, but it does not need to. The 3D model minimally needs to be sufficiently representative of a very small Late Triassic dinosauriform (ideally from Argentina), and it succeeds at this regardless of any of the above concerns. All of the issues we raise above are, in principle, testable, modifiable and reusable with our model provided here.

### 3.3 Body shape and segment dimensions of the 3D model

Our model (Figure 2b; Table 5) is 0.46 m long, with 0.12 m long hindlimbs and an estimated 0.134 kg body mass; with a COM very close to the hip joints (0.0027 m cranially; 0.011 m ventrally). The COM position is well positioned to enable habitual bipedalism, making it easy to position the pedes underneath the COM (e.g., Bishop, Cuff, & Hutchinson, 2021). Sensitivity analysis of trunk segment mass, achieved by increasing that segment's mass by 20%, shifted the whole‐body COM cranially to a position of 0.0048 m along the x‐axis (= 78% change). However, this change still is only 1% of total body length and 6.3% (vs. 3.5% originally) of gleno‐acetabular distance, so the COM remains very close to the hip joint.

Allen et al. (2013; also in Bishop et al., 2020) reconstructed the BSPs for “*Marasuchus*” using the NHMUK R 14101 cast, and Table 5 uses those data, with BSP data for *Coelophysis* (Bishop, Cuff, & Hutchinson, 2021) for comparison; with all values shown relative to body mass or segment length. This comparison is useful first because it reveals how much specimen choice and reconstruction of one taxon (*Lagosuchus* vs. “*Marasuchus*”) impacts BSP estimates, and second because *Lagosuchus* and *Coelophysis* are good rough proxies for the body shapes of Late Triassic non‐dinosaurian Dinosauriformes and Theropoda, respectively, so they allow inferences about key evolutionary changes of body plan (e.g., Allen et al., 2013; Macaulay et al., 2023). Compared with the cast of “*Marasuchus*” (which is about twice as heavy and has hindlimbs ~34% longer; similar to PVL 3871 vs. 3870; Sereno & Arcucci, 1994), our *Lagosuchus* model has a relatively heavier tail and combined head and neck, but lighter thorax (“body” and “trunk”) and forelimbs and hindlimbs. The relative craniocaudal (for axial) or proximodistal (for appendicular) segmental COMs (i.e., along x‐axis in our model) of the tail and thorax are more cranially located (differences in the hindlimb COMs are due to different modelling); but the whole‐body COM is far more caudally located in our model. These differences can be ascribed mainly to the gracile and foreshortened tail in the NHMUK PV R 14101 reconstruction (see above; and Figure 2 vs. Figure S1); and to slightly different modelling methods and subjective decisions by different investigators over more than a decade. Compared with *Coelophysis*, our *Lagosuchus* model has a relatively heavier tail, head and neck (slightly), forelimbs, and hindlimbs (slightly; attributable to all but the thigh), but lighter thorax. The relative segmental COMs of the hindlimb are more cranially/proximally located; vs. more caudally positioned in the tail (slightly) and thorax, and again the whole‐body COM is far more caudally located. These differences diverge from general trends for the evolutionary transition from early dinosauriforms to theropods (e.g., heavier tails and lighter forelimbs, overall resulting in a caudal shift—or at least stasis—of the COM; Allen et al., 2013; Macaulay et al., 2023). More models of disparate taxa around this transition are needed to resolve this issue.

### 3.4 Estimates of muscle architecture

#### 3.4.1 Method 1

The AAs of *Lagosuchus* are best understood in relative context, versus each other and compared with other extinct Dinosauriformes. Percentages of total muscle origin AA (or CFL insertion AA) are in Table 6. The 5 largest AAs are (from largest to smallest) for FMTE, FMTI, PIFE1, PIFE2, and FL; although the CFL origin AA certainly dwarfs all of these. *Coelophysis bauri*, which has a much larger pelvis overall relative to the body, and different hindlimb bone proportions, has total AAs (Table 7 in Cuff et al., 2023) ~26 times those of *Lagosuchus*, and its CFL origin AA is of similar relative size at 21 times that of *Lagosuchus*. The five largest AAs other than CFL in *Coelophysis* (again in order) are FMTE, PIFE1, CFB, FMTI and ITC. These reflect the expansion of the ilium (i.e., brevis shelf for hip extensor CFB and cranial iliac blade for hip flexor/abductor ITC) in neotheropods such as *Coelophysis*. Similarly, the reduced fibula (typical of theropods) in *Coelophysis* vs. *Lagosuchus* relates to the relatively larger FL in the latter. Allen et al. (2013) reconstructed the masses of the CFL (relative to mean body mass) in the two taxa as 3.3 times greater in *Coelophysis*. This difference (0.9% body mass vs. 3% body mass in *Coelophysis*) resulted from a small volume of CFL in *Lagosuchus* (their “*Marasuchus*”). Notably, the mean tail mass in their reconstruction of *Coelophysis* was only 1.4 times larger than their *Lagosuchus*. Conspicuously, the tail was shorter (Figure 2 vs. Figure S1) and its flesh outlines were ventrally abbreviated in the NHMUK PV R14101 *Lagosuchus* model. The AAs for the CFL origin in our *Lagosuchus* and *Coelophysis* models are similar: ~60%–65% total AA.

**TABLE 6:** Pelvic limb attachment areas (AAs) of muscle origins for *Lagosuchus* and *Coelophysis*: Raw data; then those data as percentages of total AA (*Coelophysis* data from Cuff et al., 2023).

Muscle	*Lagosuchus*		*Coelophysis*	
	AA mm^2^	% total AA	AA mm^2^	% total AA
IT1‐3	4.1	0.28	330	1.00
FMTE	71.3	4.92	1793	5.46
FMTI	58.4	4.03	1145	3.49
AMB	4.1	0.29	131	0.40
ILFB	10.3	0.71	741	2.26
ITC	6.8	0.47	1004	3.06
IFE	12.5	0.86	671	2.04
PIFI1	20.0	1.38	64	0.20
PIFI2	18.1	1.25	751	2.29
ADD1	9.3	0.64	143	0.44
ADD2	8.7	0.60	251	0.76
PIFE1	57.2	3.95	1191	3.63
PIFE2	41.6	2.87	852	2.59
PIFE3	17.5	1.21	222	0.68
ISTR	18.9	1.30	112	0.34
CFB	24.6	1.70	1171	3.57
CFL	942.7	65	19,560	59.59
FTE	10.2	0.71	363	1.11
FTI1	3.9	0.27	207	0.63
FTI3	6.7	0.46	73	0.22
GE	4.1	0.29	87	0.27
GI	8.7	0.60	341	1.04
FDL	16.0	1.10	528	1.61
FHL	2.0	0.14	68	0.21
EDL	20.4	1.41	590	1.80
TA	1.8	0.12	41	0.13
FL	35.4	2.44	297	0.90
FB	12.2	0.84	73	0.22
AHD	1.6	0.11	24	0.07
TOTAL	1449	100	32,823	100

**TABLE 7:** Comparison of muscle attachment areas (AAs in mm^2^ normalised by body mass^0.67^) between archosaurs. AAs were measured directly for *Crocodylus niloticus* and *Eudromia elegans*; or estimated for extinct taxa using “muscle maps” and osteological correlates via the EPB (see Methods and Cuff et al., 2023). Muscle abbreviations are in Table 4; avian muscle homologies are listed in Cuff et al. (2023). Empty cells = missing data. “0” values = muscle is absent. “CFL ins.” = insertion of the CFL (origin data are discussed in the text). IFE and ITC origin AAs listed for *Crocodylus* are for the IF muscle; not two separate values.

Muscle	*Crocodylus*	*Lagosuchus*	*Coelophysis*	*Eudromia*
IT1‐3	53	16	59	224
FMTE	73	274	320	322
FMTI	434	224	204	523
AMB	33	16	23	5
ILFB	25	40	132	53
FTE	18	39	65	157
FTI1	12	15	37	0
FTI3	5	26	13	26
ITC	28	26	179	598
IFE	28	48	120	54
PIFI1	238	77	11	0
PIFI2	719	70	134	91
ADD1	43	36	26	36
ADD2	12	33	45	36
PIFE1	591	220	213	0
PIFE2	484	160	152	571
PIFE3	237	67	40	0
ISTR	114	73	20	358
CFB	39	95	209	124
CFL insertion	41	40	19	0
GL	18	16	16	27
GM	24	33	61	105
FDL	140	61	94	322
FHL	5	8	12	
EDL	7	79	106	198
TA	70	7	7	74
FL	65	136	53	55
FB	27	47	13	
AHD		6	4	0

It is also interesting in an evolutionary context for Archosauria to compare our AA data for the two Dinosauriformes addressed above with those from digitised muscle AAs in extant Pseudosuchia: Nile crocodiles *Crocodylus niloticus*, and extant Dinosauriformes: Elegant‐crested tinamous *Eudromia elegans* (Cuff et al., 2023). These data (made dimensionless for clearer comparison) are shown in Table 7. The five largest AAs (not counting the huge CFL in non‐avian taxa) in the crocodiles are PIFI2, PIFE1, PIFE2, FMTI, and PIFI1; in the tinamous (abbreviations for homologous muscles) they are ITC, PIFE2, FMTI, ISTR and FMTE (tied with FDL, which in birds is only partly homologous with the FDL of non‐avian archosaurs; Hattori & Tsuihiji, 2020). There are interesting differences in these data that match skeletal transformations in traits that are roughly ancestral archosaurian ones (e.g., Hutchinson, 2001a, 2001b; see also Rhodes et al., 2021) retained in *Crocodylus* vs. derived in Dinosauriformes. For example, the dimensionless FMTE knee extensor AA is about four times larger in all Dinosauriformes vs. *Crocodylus*, largely because the IF insertion occupies more of the lateral femoral shaft in *Crocodylus*, constraining the FMTE origin's size. Assuming that AA directly correlates with PCSA in these muscles across Archosauria, a broader implication, to our knowledge not noted in prior studies, is that proximal concentration of the IF (= ITC + IFE) insertion likely enabled the FMTE muscle size to enlarge—and transformation of the IF muscle complex's insertion from a large ‘fleshy’ one into two tendinous ones still seems to have enabled the latter muscle complex to remain large (and expand across dinosauriform evolution). Furthermore, because the PIFI2 hip flexor group of *Crocodylus* has a very expansive ‘lumbar’ origin, it is far larger than its homologues in Dinosauriformes (Table 7; AA/body mass^0.67^ of 719 vs. 70, 134 and 91 in *Lagosuchus*, *Coelophysis* and *Eudromia*). This greater size is in contrast to the unexceptional PIFI2 hip flexion moment arm in many pseudosuchians vs. dinosauriforms (Cuff et al., 2022), implying relatively greater PIFI2 hip flexor moment‐generating capacity in Crocodylia (and other taxa with ‘lumbar’ vertebral origins, probably) vs. in many Dinosauriformes. Other muscle AAs are broadly similar between these taxa except that the PIFI1 is also much larger in *Crocodylus* (AA/body mass^0.67^ of 238 vs. 77 and 11 in extinct Dinosauriformes; absent in *Eudromia*) whereas the ITC + IFE group in Dinosauriformes is 2.6–23 times larger than its homologue in *Crocodylus*, the IF. Similarly, postacetabular muscles in Dinosauriformes, such as ILFB and FTE, are around two or more times larger, due to the expanded ilium. Finally, the PIFE1‐3 group is ~2–3 times larger in *Crocodylus* than the Dinosauriformes. Conspicuously, the PIFE3 is sequentially reduced (AA/body mass^0.67^ of 237 in *Crocodylus* vs. 67 in *Lagosuchus* and 40 in *Coelophysis*; absent in *Eudromia*) as expected from the reduction of the cranial side of the ischial part of the puboischiadic plate. Together, these and other similarities and differences in relative AA sizes, indeed, correspond to the expected evolutionary polarities for Archosauria; particularly expansion of the pelvis (Hutchinson, 2001a, 2002; see also Rhodes et al., 2021). Nonetheless, more data from extant taxa as well as estimates for key extinct archosaurs are needed to test these preliminary speculations on changes in muscle AAs.

#### 3.4.2 Methods 1 vs. 2

Here we compare the results of Method 1 and Method 2 for estimating PCSA (Table 8) and thus F_max_ for our *Lagosuchus* model. Somewhat unlike the results for *Coelophysis* in Cuff et al. (2023), where PCSA seemed strongly underestimated by Method 1, we find that the two methods give results that overall have some similarities (ratio of total PCSAs of Method 2 vs. 1 = 1.31). That ratio drops to 1.10 if the very short‐fibred FB muscle in Method 2 is ignored, because it is assigned an implausibly large PCSA and F_max_, twice as large as the next largest muscle, FL; surely due to their very small average moment arms around the ankle producing very small estimated fibre lengths. However, many values (as % total PCSA) are quite different—notably, Method 2 gives greater PCSA estimates for the IT1 (3.08 times), EDL (1.87 times), ITC and IFE (>1.8 times); with 9 out of 22 muscles having ratios >1.5 and 11 more with ratios >1. Relative PCSAs for Method 2 vs. 1 are <1 (i.e., relatively smaller) for 12 out of 32 muscles, especially the CFL (0.11), GE (0.31) and FDL (0.38). Considering Method 2's potential overestimates for FB (and maybe FL) and underestimate for CFL, the two methods tend to give more similar results than it may seem.

**TABLE 8:** Comparison of pelvic limb muscle PCSA estimates for *Lagosuchus*. AAs from Table 7 were used to estimate PCSAs (column “PCSA”) following Cuff et al. (2023), then those data are shown as percentages of total PCSA; then portrayed as PCSA ratios for Method 2 (PCSAs calculated for Method 2 as per the Methods section) vs. Method 1. We used the “one size fits all” equation (PCSA in mm^2^ = (3 × 10^−7^ (AA) + 5 × 10^−5^)) from Cuff et al. (2023) for estimating the AMB and CFL PCSAs for both taxa because the muscle‐specific equations curiously gave negative values in *Lagosuchus*, and that equation for the GI's PCSA as it gave values more plausibly close to those in extant archosaurs; and the same equation for the CFL origin (not insertion as in Cuff et al., 2023).

Muscle	PCSA	% total PCSA	Ratio Method 2 vs. 1
IT1	17.4	0.94	3.08
IT2	59.3	3.21	0.85
IT3	40.6	2.20	1.79
FMTE	59.4	3.22	1.04
FMTI	55.9	3.03	1.07
AMB	41.4	2.24	0.82
ILFB	43.0	2.33	1.04
ITC	42.1	2.28	1.81
IFE	43.6	2.36	1.85
PIFI1	45.6	2.47	1.06
PIFI2	45.1	2.45	1.44
ADD1	42.7	2.32	1.02
ADD2	42.6	2.31	1.16
PIFE1	55.6	3.01	0.53
PIFE2	51.4	2.79	0.70
PIFE3	44.9	2.44	1.34
ISTR	77.9	4.22	1.41
CFB	81.9	4.44	0.60
CFL	293.5	15.91	0.11
FTE	73.6	3.99	0.64
FTI1	41.3	2.24	0.63
FTI3	39.6	2.15	1.73
GE	121.1	6.56	0.31
GI	42.6	2.31	1.63
FDL	44.5	2.41	0.38
FHL	26.7	1.45	0.95
EDL	55.7	3.02	1.87
TA	45.7	2.48	0.58
FL	86.0	4.66	1.65
FB	43.5	2.36	6.33
AHD	40.7	2.20	1.13
TOTAL	1845	100	
MEAN (S.D.)			1.31 (1.09)

### 3.5 Muscle moment‐generating capacities

Next, using Method 2's data for muscle architecture (Table S1; Method 1's data should give approximately similar results; as per Tables 6 and 8), we compare (non‐dimensionalised) maximal isometric “antigravity” muscle moment‐generating capacities around the hindlimb joints in our musculoskeletal models of *Lagosuchus* vs. *Coelophysis* (Table 9). Note that this comparison involves placing the models of these taxa into similar poses (see Methods). Allometric scaling of posture‐related moment arms (e.g., Biewener, 1989) has not been considered and might cause more differences. ROMs are similar for our models of the two taxa (Figure 3 vs. Figure 3 in Bishop, Cuff, & Hutchinson, 2021)—the main differences are ~45% greater non‐sagittal hip ROMs in *Lagosuchus* (due to the less restricted acetabulum), which are not considered in Method 2. Under these and other assumptions involved, relative to its size, *Lagosuchus* may have been able to produce ‘antigravity’ muscle moments on average relatively 5.08 times larger vs. *Coelophysis*; principally due to its muscles acting around the same joints being relatively 4.54 times stronger. Under isometry, one would expect that lengths scale with lengths^1.00^, and lengths scale with body mass^0.333^. As F_max_ is proportional to area, it would be expected to scale with mass^0.667^; we therefore expect that F_max_, when normalized for body weight, will scale with body mass to the power of 0.667–1.0 = mass^−0.333^. Simple application of scaling principles from our *Lagosuchus* to *Coelophysis* models suggests that the muscle moment arms have little scaling influence on the moment‐generating capacities, because they are only on average relatively 1.07 times larger in *Lagosuchus*. However, this similarity of average values masks underlying differences in antigravity muscle moment arms (see Table 9 ratios for *Lagosuchus* vs. *Coelophysis*). There is a notably smaller ratio (0.879) of muscle moment arms for the hip extensors that is plausibly consistent with major differences in pelvic morphology (e.g., relatively larger pelvis that shifts muscle paths further away from the hip in *Coelophysis* and other dinosaurs; Allen et al., 2021; Carrano, 2000). The muscles acting around the more hinge‐like distal hindlimb joints also vary in their moment arm ratios for *Lagosuchus* vs. *Coelophysis*, being greater (knee: 1.25; MTP 1.50) or smaller (ankle: 0.729), which may either reflect real differences in musculoskeletal morphology or be an artefact of modelling these joints' JCSs and/or muscle paths. Nonetheless, the difference in F_max_ noted above is trivially different from isometric scaling (slope of −0.330; isometric slope = −0.333). This finding of isometric scaling for total ‘antigravity’ muscle moment‐generating capacity is due to similar morphology (i.e., actual similarity) and geometry (via a similar model‐building approach) in the models, rather than using similar limb poses. However, we caution that our focus has only been on these muscles and certain metrics of hindlimb biomechanics; and for just two dinosauriform taxa. Furthermore, Method 2 involves computing moment arms (via musculotendon length change calculations) to estimate fibre lengths (and thereby F_max_) and moment arms are then compared in our moment‐generating analyses, so moment arm inaccuracies might be compounded. Across all hindlimb muscles here (Table S2), fibre lengths scale with slopes of 0.349 (if compared to body mass; isometry = 0.333) or 1.135 (if compared to hindlimb length; isometry = 1.00), so via Method 2 *Lagosuchus* generally has somewhat shorter muscle fibres. *Lagosuchus* has slightly longer hindlimbs vs. *Coelophysis* (allometric slope of 0.307) but this does not seem to influence averaged muscle moment arms, either due to similar muscle‐tendon unit path constraints in the model or more proximal muscle insertions. Thus there is much more to be learned about the scaling of locomotor function in Dinosauriformes.

**TABLE 9:** Comparison of components of dimensionless maximal isometric ‘antigravity’ muscle moment‐generating capacities for models of the hindlimbs of *Lagosuchus* and *Coelophysis*. Total dimensionless forces (F_max_), average moment arms, and moments are shown for the four main hindlimb joints, then a ratio of those averages for (*Lagosuchus* / *Coelophysis*), and then the average value for these ratios is provided. Moments were taken as absolute values and non‐dimensionalised by dividing by body weight (= mass * 9.81 m s^−2^) and hindlimb length, and were calculated from model moment arms in the pose used (see Figure 2B and Bishop, Cuff, & Hutchinson, 2021; semi‐crouched pose) times F_max_ values derived from PCSAs (Table S1; and Method 2).

Joint	Total F_max_			Average moment arms			Total moments		
	*Lagosuchus*	*Coelophysis*	Ratio	*Lagosuchus*	*Coelophysis*	Ratio	*Lagosuchus*	*Coelophysis*	Ratio
Hip	130	33.9	3.83	0.0511	0.0645	0.792	6.04	1.66	3.63
Knee	76.2	13.6	5.61	0.0348	0.0285	1.25	2.61	0.296	6.03
Ankle	76.9	17.5	4.40	0.0145	0.0199	0.729	0.961	0.230	4.17
MTP	9.69	2.24	4.32	0.0162	0.0108	1.50	0.157	0.0243	6.47
		average	4.54		average	1.07		average	5.08

### 3.6 Scope for improvements of the 3D model

Key assumptions of our model hierarchically impact its output data (see, for example, Bishop, Cuff, & Hutchinson, 2021 for elaboration). First, our composite skeletal model relies on the identifications, scaling, and replacement of missing elements from various sources (Aim 1; Tables 1, 2; Figures 7, 8, 9, 10, 11). We have noted cases (e.g., the forelimbs) where more study of the taxonomic assignments of elements is needed. Of course, further discovery of more complete “lagosuchid” remains; or additions of better identified existing museum specimens (for example, MCZ specimen numbers 4137, 4346, 9483 and 101667 catalogued as *Lagosuchus talampayensis*); could improve our model. Second, we used that skeletal model to estimate BSPs (Table 5; Figure 2b), using methods that have some basis in data from extant taxa (Allen et al., 2009) but that basis deserves expansion. Alternatively, other methods such as convex hull estimates (Macaulay et al., 2023; Sellers et al., 2012) could be applied, although the axial skeleton (especially ribcage) is presently too incompletely known for that purpose. Either of these steps could improve our model. Third, we have used current ‘best practice’ approaches (Gatesy et al., 2022) to estimate our JCSs (Figure 2a), compatible with those used for studies of other archosauriform taxa. Yet we have acknowledged that our usage of these JCSs and articular morphology for estimating ROMs (Figure 3) is subjective and simplistic. The ROM data are necessary as a basic foundation for our model of whole‐limb function, but are not sufficient for conclusive inferences at the level of individual joint mobility and its evolution (e.g., Bishop et al., 2023; Demuth et al., 2020; Kambic et al., 2014; Manafzadeh et al., 2021, 2024; Manafzadeh & Gatesy, 2021). However, because our JCS and skeletal geometry are compatible with these more sophisticated methods, they can be used in future studies to refine our model or make such inferences with plausible precision. Joint mobility estimates could be further refined by including ligaments and similar passive structures (e.g., Manafzadeh & Padian, 2018), which also can supplement muscle moments to support joints. Fourth, we have employed fundamental methods for pelvic limb muscle reconstruction (Table 4; Figures 4, 5, 6) dating back almost three decades (e.g., Hutchinson, 2001a, 2001b, 2002; Witmer, 1995) yet more data‐driven approaches for estimating muscle paths, such as geometric formulations of the relationships of those paths with underlying skeletal geometry in extant archosaurs (e.g., via contrast staining and computed tomography; Allen et al., 2017; Bishop, Michel, et al., 2021; Wiseman et al., 2021), could transform those fundamental methods and thereby improve our model. Fifth, estimation of limb muscle architecture in any extinct taxa remains in an early, tentative stage with multiple potential methods still in usage (discussed in Cuff et al., 2023; also see Charles et al., 2022 for some concerns). We applied two data‐driven approaches (Methods 1 and 2; Figure 6; Tables 6, 7, 8, 9; Data S1, S2) but noted others that exist, and the need for more data for and checking of existing methods (see also Bates and Falkingham 2018; Bishop, Michel, et al., 2021; Bishop, Wright and Pierce, 2021; Charles et al., 2022; Demuth et al., 2022). This step is tremendously important for using models such as ours in higher‐level inferences such as simulations (e.g., Bates et al., 2021; Bishop, Cuff et al., 2021; Bishop, Falisse et al. 2021; Demuth et al., 2023; Sellers et al., 2009, 2013, 2017). It remains a crucial frontier. Nonetheless, we contend that our model is a reasonable representation of a Late Triassic dinosauriform (Figure 1) such as *Lagosuchus talampayensis*, based on our current understanding of these animals. As we have taken cautious steps to maintain reliability while keeping limitations of our methods in mind, our model provides a useful foundation for biomechanical analysis of this organism, and for placing it into a broader evolutionary context of the history of archosaur locomotion (e.g., Cuff et al., 2022; Hutchinson & Gatesy, 2000).

## 4 CONCLUSIONS

We have achieved our five aims, finding that (1) there seem to be hitherto unappreciated skeletal elements curated with the PVL *Lagosuchus talampayensis* material that are not from that taxon, including a previously unrecognised silesaurid pelvis—yet specimen PVL 3870 and some other specimens remain reasonably assignable to *L. talampayensis*; (2) these elements helped us to form what we feel is the best currently feasible model approximating the skeletal dimensions of *L. talampayensis*; (3) similarly, those skeletal dimensions enable a more rigorous reconstruction of 3D body shape than in our prior analyses (mainly Allen et al., 2013), and generally match hypothesised major transformations of the dinosauriform to theropod body plans in the Triassic period; (4) together, all of these data form a foundation for a 3D musculoskeletal model (of the pelvic limb muscles) of *L. talampayensis* for which various methods of estimating muscle architecture can be applied; and (5) attachment area‐based estimates of muscle force capacity in *L. talampayensis* are roughly similar to those from another popular method, for reasons not yet understood; but those areas themselves, by their quantitative nature, help to clarify how muscle sizes transformed across Archosauria, in relation to changes of the morphology of the pelvic limb skeleton. The resulting model not only is valuable for addressing biomechanical hypotheses, but also evolutionary questions, particularly as *L. talampayensis* is widely agreed to be at a phylogenetically pivotal point in dinosauriform phylogeny for gauging evolutionary polarity from early Archosauria to Dinosauria. Our process of constructing the composite model should be a useful example for how to make such models from problematic taxa. During this process, we also illuminated a problem with the taxonomic assignment of various skeletal elements of certain specimens that poses a conundrum for understanding the ‘real’ *Lagosuchus* and other early dinosauriforms, and a challenging opportunity to reveal new archosaurian taxa from the Late Triassic of Argentina.

## FUNDING INFORMATION

This study was supported by funding from a ERC Horizon 2020 Advanced Investigator Grant (#695517) to J.R.H.

## CONFLICT OF INTEREST STATEMENT

The authors declare that they have no conflicts of interest.

## Supporting information


Figure S1.

Table S1.
**Table S2**.

Data S1.

Data S2.

## Data Availability

OpenSim model files are at Figshare (doi: 10.6084/m9.figshare.25955887): https://figshare.com/s/069870861af7bcc61798. Raw CT scan, mesh, and high‐resolution photograph files are at MorphoSource: [https://www.morphosource.org/projects/000669434/temporary_link/Gy6ks6GAykk6CkHNG9dRXfoS].
