# Supplementary figures and images for: Hindlimb biomechanics of Lagosuchus talampayensis (Archosauria, Dinosauriformes), with comments on skeletal morphology
Source: J Anat. 2024 Dec 4;246(6):948–73. doi: 10.1111/joa.14183 (PMC12079757; doi:10.1111/joa.14183)

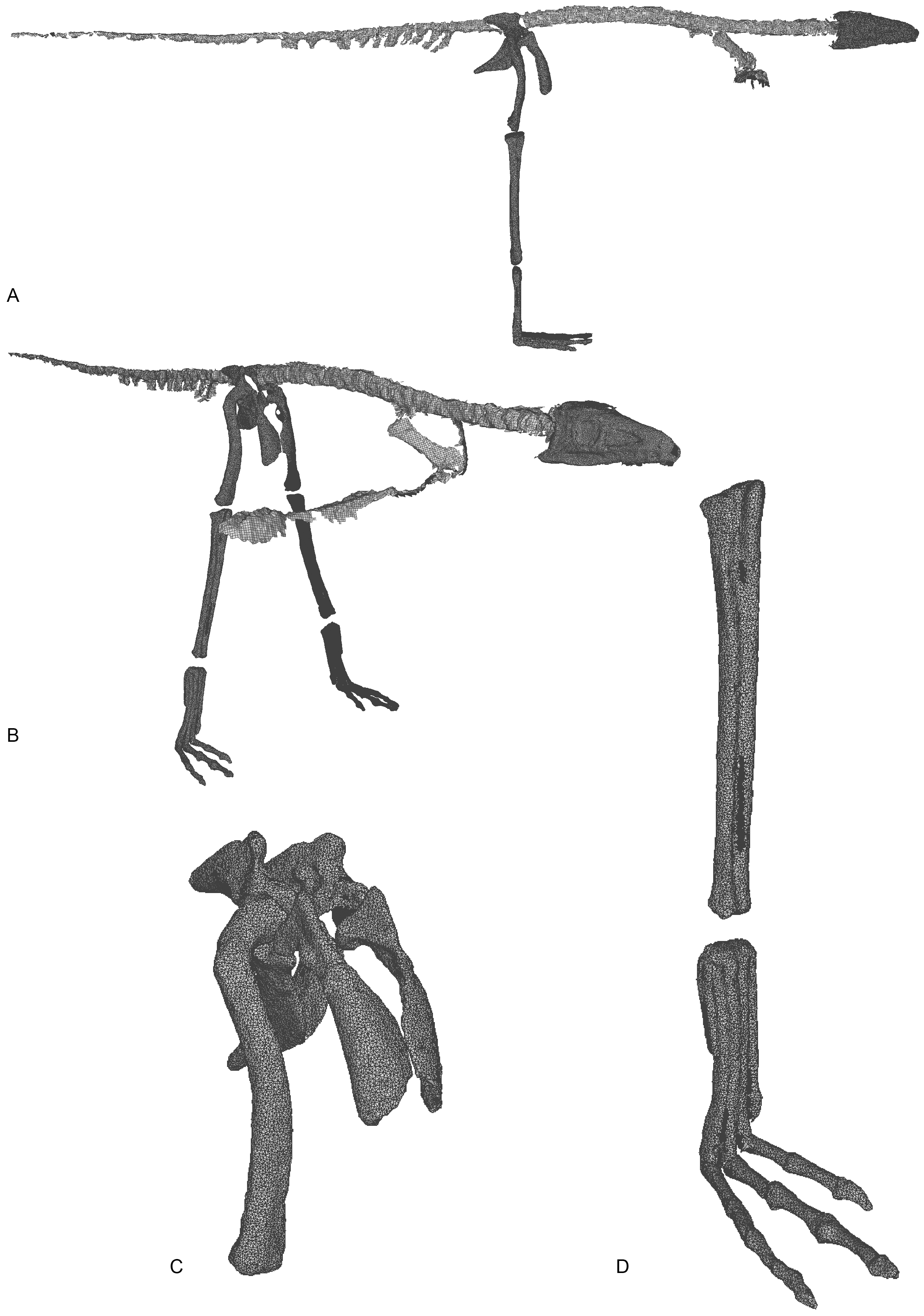

Supplement: Supplementary file 1 — Figure S1. [file JOA-246-948-s001.jpg]
