# Supplementary material for: Hindlimb biomechanics of Lagosuchus talampayensis (Archosauria, Dinosauriformes), with comments on skeletal morphology
Source: J Anat. 2024 Dec 4;246(6):948–73. doi: 10.1111/joa.14183 (PMC12079757; doi:10.1111/joa.14183)
Supplement: Supplementary file 3 — Data S1. [file JOA-246-948-s004.doc]

**Supplementary Information for:**

**Hindlimb biomechanics of *Lagosuchus talampayensis* (Archosauria, Dinosauriformes), with comments on skeletal morphology**

Alejandro Otero^1^, Peter J. Bishop^2,3,4^ and John R. Hutchinson^2^

^1^ CONICET. División Paleontología de Vertebrados (Anexo Laboratorios), Museo de La Plata, Paseo del Bosque s/n, La Plata 1900, Argentina. [alexandros.otero@gmail.com](mailto:alexandros.otero@gmail.com)

^2^ Structure and Motion Laboratory, Department of Comparative Biomedical Sciences, Royal Veterinary College, North Mymms, Hatfield, AL9 7TA, UK.

^3^ Museum of Comparative Zoology, Department of Organismic and Evolutionary Biology, Harvard University, Cambridge, MA 02138, USA. [pbishop@fas.harvard.edu](mailto:pbishop@fas.harvard.edu)

1. Geosciences Program, Queensland Museum, Brisbane, Queensland 4011, Australia.

**Reconstructing muscle character states in *Lagosuchus talampayensis***

We first coded our operational taxonomic units (i.e., terminal taxa) for the muscle character states. In the case of *Lagosuchus*, it was coded as part of “Lagosuchidae”. We then used the maximum parsimony method in Mesquite 3.81 software for ancestral state reconstruction, to obtain character states at each node in the phylogeny, following this workflow:

- For visualization and interrogation of individual characters across phylogeny, using Analysis: Tree > Trace character history > Parsimony ancestral states.
- For reconstruction of ancestral states at all nodes, using Analysis: Tree > Trace all characters > Parsimony ancestral states to tabulates the reconstructed states for all characters for all nodes; copy-pasted into an Excel spreadsheet for further analysis.

For *Lagosuchus*, we used a revised dataset and ‘consensus’ tree based on those used in Bishop et al. (2021a), with scored states for Lagosuchidae, and retrieved the below reconstructed states for that or the Dinosauriformes node. ‘01’, ‘012’, ‘12’ and ‘02’ signify an ambiguous state at this node, with the parenthetical value denoting the final resolved state:

| character | 1 | 2 | 3 | 4 | 5 | 6 | 7 | 8 | 9 | 10 |
| --- | --- | --- | --- | --- | --- | --- | --- | --- | --- | --- |
| state | 1 | 0 | 1 | 0 | 1 | 1 | 0 | 0 | 0 | 0 |
| character | 11 | 12 | 13 | 14 | 15 | 16 | 17 | 18 | 19 | 20 |
| state | 1 | 0 | 0 | 0 | 0 | 0 | 1 | 2 | 3 | 1 |
| character | 21 | 22 | 23 | 24 | 25 | 26 | 27 | 28 | 29 | 30 |
| state | 1 | 1 | 1 | 0 | 0 1  (1) | 2 | 0 1 (0) | 0 | 0 | 0 1 2 (2) |
| character | 31 | 32 | 33 | 34 | 35 | 36 | 37 | 38 | 39 | 40 |
| state | 1 2 (2) | 2 | 0 1 (1) | 1 2 (2) | 0 | 0 2 (0) | 1 | 2 | 2 | 0 1 (0) |
| character | 41 | 42 | 43 | 44 | 45 | 46 | 47 | 48 | 49 | 50 |
| state | 1 | 0 1 (0) | 0 1 (0) | 1 | 1 | 1 | 1 | 1 | 1 | 1 |
| character | 51 | 52 | 53 | 54 | 55 | 56 | 57 | 58 | 59 | 60 |
| state | 1 | 1 | 1 | 0 | 2 | 0 | 0 | 1 | 1 | 1 |
| character | 61 | 62 | 63 | 64 | 65 | 66 | 67 | 68 | 69 | 70 |
| state | 1 | 1 | 2 | 0 1 (0) | 0 | 1 | 1 | 1 | 0 1 (0) | 0 1 (0) |
| character | 71 | 72 | 73 | 74 | 75 | 76 | 77 | 78 | 79 | 80 |
| state | 1 | 0 1 (0) | 1 | 1 | 0 | 1 | 0 | 0 | 0 | 1 |
| character | 81 | 82 | 83 | 84 | 85 | 86 | 87 | 88 | 89 | 90 |
| state | 1 | 1 | 1 | 0 | 0 | 1 | 0 | 0 | 0 | 1 |
| character | 91 | 92 | 93 | 94 | 95 | 96 | 97 | 98 | 99 | 100 |
| state | 1 | 1 | 0 | 0 | 0 | 0 | 0 | 1 | 2 | 2 |
| character | 101 | 102 | 103 | 104 | 105 | 106 | 107 |  |  |  |
| state | 0 | 0 | 0 | 0 | 2 | 0 | 0 |  |  |  |

The following (mostly still-ambiguous) character states for *Lagosuchus* were then resolved as follows (“inference” = level of inference from Witmer (1995)):

Character 7: this was coded as 0&1 for Lagosuchidae, but was coded as 0 for *Lagosuchus* by Hutchinson (2002); and reconstructed for Dinosauriformes here; which we retain here (II′ inference).

Character 13: this was coded as 0 for Lagosuchidae.

Character 25: there is no ‘cuppedicus fossa’ on the preacetabular ilium of *Lagosuchus*, but there is some reduction of the puboischiadic plate, and so we adopt the Level II inference that the PIFI1 originated from the medial ilium or proximodorsal puboischiadic plate (i.e., state 1).

Character 27: there is no evidence of two discrete PIFI1 insertion scars on the femur of *Lagosuchus*, (= two ‘avian’ muscles), and so we infer a single head to this muscle (state 0; II′ inference).

Characters 30, 31, 33: as per Bishop et al. (2021a), we infer that the PIT was absent in *Lagosuchus* based on the distal expansion of the pubic and ischiadic symphyses (and reduced puboischiadic plate), and so reconstruct these characters as states 1 (character 33) or 2 (II inference).

Characters 34-39: as per Bishop et al. (2021a), we reconstruct only two heads for the FTI complex, homologous to FTI3/FCLP and FTI1/absent of Crocodylia/Aves, respectively, (character 34 = state 2, II inference). Clear osteological correlates on the ischium are lacking for either muscle, probably on account of the very small size of *Lagosuchus* (character 35 = state 0, II′ inference) although the caudalmost proximal part of the ischium is modestly expanded behind the acetabulum, and may signal where the FTI3 originated from (character 37 = state 0, II′ inference). Osteological evidence of insertion is also absent, but we are confident of primary (if not sole) insertions on the caudomedial (FTI1) to caudal (FTI3) proximal tibia (characters 36 and 39, states = 0 and 2, I′ inference).

Character 40: we deem the presence of a secondary tendon of insertion to be of minor importance in a mechanical sense, and so is omitted here (character 40, state 0, II′ inference).

Characters 42, 43: we follow the same logic as that presented by Bishop et al. (2021a), reconstructing both characters as state 0 for *Lagosuchus* (II′ inference).

Character 62: scored as 1&2 for Lagosuchidae, but scored as 1 for *Lagosuchus* by Hutchinson (2002); and reconstructed for Dinosauriformes here; which we follow.

Character 64: following Bishop et al. (2021a), we assume an absence of ‘avian’ traits (even more justifiable for *Lagosuchus* than *Coelophysis*), and reconstruct only two parts to the gastrocnemius (state 0, II′ inference). Note that this contrasts with studies such as Piechowski and Tałanda (2019), who reconstructed a more ‘avian’ state here and for some other traits.

Character 66: scored as 1 for Lagosuchidae (including *Lagosuchus*), which retain a small calcaneal tuber; *Lagosuchus* also possesses a second caudally directed projection on distal tarsal IV (Sereno and Arcucci, 1994).

Characters 69, 70: as above, gastrocnemius pars intermedia is absent in *Lagosuchus* (state 0, II′ inference).

Character 72: Bishop et al. (2021a) conservatively assumed that subdivision of the digital flexor mass on the avian stem lineage occurred at a more crownward node than Neotheropoda, and we therefore reconstruct only the flexor digitorum longus and flexor hallucis longus here (state 0, II′ inference).

Following Hattori and Tsuihiji (2020), we revised some homologies and character codings from those of Hutchinson (2002) and Bishop et al. (2021a), as follows (all states were coded as unambiguous for *Lagosuchus*):

Character 73: we place the FHL origin as extending from the caudolateral distal femur near the GE origin (state 0) to be expanded onto the lateral cnemial crest of the tibia, fossa flexoria, and proximal fibula (state 1; II′ inference).

Character 74: considering the digitigrade pes and correspondingly reduced tarsals and some digits, we infer absence of the FDB muscle, as in birds (state 1; II inference).

Character 75: we infer a plesiomorphic FDL origin from the proximomedial fibula’s shaft (state 0; arguably II not II′ inference); considering the relatively plesiomorphic morphology of the fibula.

Character 76: as per character 74, and the loss of digit V, we infer derived digital flexor insertions solely onto the flexor tubercles of the pedal unguals (mainly II-IV for FDL; I-IV for FHL) (state 1; II inference).

Characters 77, 78, 85: these pertain to the absence/presence of other derived ‘avian’ traits (e.g., tibial cartilage; ossified FDL tendon, which we conservatively score here as absent (normally state 0; II′ inference); most or all of these would have negligible impacts on our biomechanical model either way.

Character 79: we infer the FHL’s insertion on digit I to be caudal, as the hallux is not retroverted (state 0; II inference).

Character 80: we consider the FHB to be absent, based upon the relatively immobile distal tarsals and possibly less mobile metatarsal I (state 1; II inference); thus characters 81 and 82 are state 1 (absent).

Character 83: importantly, Hattori and Tsuihiji (2020) revised homologies with this muscle and the EDL. We reconstruct the TA origin as still mainly coming from the craniolateral side of the distal femur, but also expanded onto the lateral cnemial crest, which is enlarged (state 1; II inference).

Character 86: the EDL origin is reconstructed as from the lateral side of the cremial crest (based on its expansion); distal to the TA origin; and the cranial tibial shaft (state 1; II inference).

Character 87: as per discussion of the EDB for characters 90-92 below, we reconstruct the EDL insertion in its derived position onto the dorsal surfaces of the distal pedal phalanges (state 1; II inference).

Characters 88, 89: our analysis does not go to the level of complexity of hallucal joints, so we do not reconstruct this muscle in our model; but based on lack of hallucal retroversion its position would be plesiomorphic (from cranial side of metatarsal I to dorsal surfaces of the phalanges; II inference).

Characters 90-92: because *Lagosuchus* has an ‘advanced mesotarsal’ ankle, reducing tarsal mobility, we infer ‘absence’ of the EDB muscle (and thus fusion to the distal EDL; Hutchinson, 2002; Hattori and Tsuihiji, 2020) (state 1; II inference). We assume that the muscle was ‘lost’ when the pes of avemetatarsalians became functionally tridactyl and tended to have less mobility in directions other than flexion–extension.

Character 93: we reconstruct the ‘pronator profundus’ (PP1 and PP2) (e.g., tibialis posterior, interosseous cruris and/or popliteus; PP2 is more similar to what is often called the popliteus in birds, whereas PP1 is more similar to the typical non-avian interosseous) as present (state 0; II inference), based on the expansive distal fibular shaft.

Character 94: we infer the PP2 origin from the caudomedial fibular shaft (state 0; II inference).

Character 95: we place the PP2 insertion onto the caudolateral side of metatarsal I and the process of distal tarsal IV, considering the more plesiomorphic morphology of these (state 0; II inference).

Characters 96, 97: we do not model tibia-fibula mobility here, but the PP1 muscle should have extended from the caudolateral tibial shaft to the medial fibular shaft (state 0; II′ inference).

Character 98-100: we omit a fibulocalcaneus muscle here (states 1 or 2; II inference), because the tibia-fibula seem to have been roughly immobile at the proximal tarsus, and the calcaneal tuber is strongly reduced.

Character 101: the AHD is presumed to have been present, evidenced by the more plesiomorphic morphology of metatarsal I and the distal fibular shaft vs. birds, which lack the AHD (state 0; II inference).

Character 102: we put the AHD origin on the distal craniolateral fibula (state 0; II inference).

Character 103: we put the AHD origin on the proximodorsal side of metatarsal I (state 0; II inference).

Characters 104-107: for the FL and FB, the plesiomorphic fibular morphology and persistence of metatarsal V (Sereno and Arcucci, 1994) support level I´ (for origin) and II (for insertion) inferences (states 0-2 depending on the character).
